# Constructing 3D interaction maps from 1D epigenomes

**DOI:** 10.1038/ncomms10812

**Published:** 2016-03-10

**Authors:** Yun Zhu, Zhao Chen, Kai Zhang, Mengchi Wang, David Medovoy, John W. Whitaker, Bo Ding, Nan Li, Lina Zheng, Wei Wang

**Affiliations:** 1 Department of Chemistry and Biochemistry, University of California at San Diego, La Jolla, California 92093-0359, USA; 2 Department of Cellular and Molecular Medicine, University of California at San Diego, La Jolla, California 92093-0359, USA

## Abstract

The human genome is tightly packaged into chromatin whose functional output depends on both one-dimensional (1D) local chromatin states and three-dimensional (3D) genome organization. Currently, chromatin modifications and 3D genome organization are measured by distinct assays. An emerging question is whether it is possible to deduce 3D interactions by integrative analysis of 1D epigenomic data and associate 3D contacts to functionality of the interacting loci. Here we present EpiTensor, an algorithm to identify 3D spatial associations within topologically associating domains (TADs) from 1D maps of histone modifications, chromatin accessibility and RNA-seq. We demonstrate that active promoter–promoter, promoter–enhancer and enhancer–enhancer associations identified by EpiTensor are highly concordant with those detected by Hi-C, ChIA-PET and eQTL analyses at 200 bp resolution. Moreover, EpiTensor has identified a set of interaction hotspots, characterized by higher chromatin and transcriptional activity as well as enriched TF and ncRNA binding across diverse cell types, which may be critical for stabilizing the local 3D interactions.

## Abstract (web summary)

The human genome is highly organized, with one-dimensional chromatin states packaged into higher level three-dimensional architecture. Here, the authors present EpiTensor that can identify 3D spatial associations from 1D epigenetic information.

## Graphical abstract

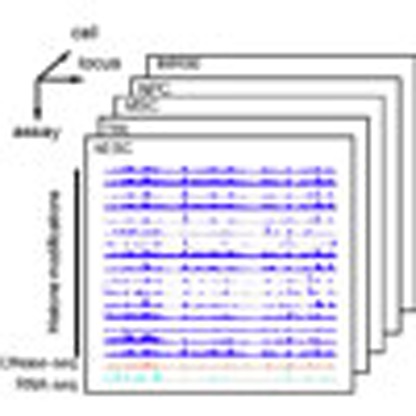

Epigenomic modifications and 3D genomic interactions are tightly associated but currently they are measured by distinct technologies and an integrative interpretation is still lacking^1^. On one hand, chromosome conformation capture (3C)-based methods, including 4C and 5C, have been developed to detect physical contacts in the 3D space^2^. However, these assays are not designed to measure 3D interactions in the entire genome. Chromatin Interaction Analysis by Paired-End Tag Sequencing (ChIA-PET) allows genome-wide measurements^3^, but its interpretation is complicated by high levels of background noise and a high rate of false negatives^4^. Moreover, ChIA-PET is restricted to interactions mediated by a preselected protein of interest. The method Hi-C allows the genome-wide detections of interactions but requires an extremely high sequencing depth to achieve high resolution^5^,^6^,^7^.

On the other hand, epigenomic assays, including chromatin modification ChIP-seq, RNA-seq and DNaseI-seq, map chromatin features along the linear genome. The current state-of-the-art analyses focus on interpreting the epigenomic data in a 1D space along the linear genome. For example, the epigenomic state of a specific locus is defined by the combination of epigenomic signals, which leads to linear segmentation of the genome; sequencing reads of epigenomic modifications are typically visualized as different tracks in a genome browser^8^,^9^. Such 1D representation and interpretation of epigenomic data neglect the important impacts of the 3D organization of chromosomes in the cell^10^. Recent efforts have started to integrate 1D and 3D data for genome annotation^11^,^12^,^13^,^14^. However, the information of 3D interactions encoded in the epigenomic data has not been effectively deciphered.

Here, we present EpiTensor, a novel unsupervised computational method to derive 3D interactions between distal genomic loci from 1D epigenomic data. EpiTensor provides a resolution significantly higher than that provided by Hi-C experiments. The current implementation provides a resolution of 200 bp, which can be further extended to even higher resolution. This work represents a systematic and unbiased attempt to infer 3D spatial patterns from 1D epigenomic data, which provides a new method complementary to Hi-C and ChIA-PET. As most of the interactions are within topologically associating domains (TAD), we constrain our analysis within TADs and show that promoter–enhancer, promoter–promoter and enhancer–enhancer associations within identified from EpiTensor are highly concordant with those from Hi-C, ChIA-PET and eQTL experiments. Furthermore, EpiTensor identified a set of interaction hotspots that have many interacting partners. We demonstrate these hotspots having higher chromatin and transcriptional activity across cell types are preferably bound by TFs and lncRNAs, and are enriched with TF motifs.

## Results

### Tensor modelling of multi-dimensional epigenomes

**Figure 1:**
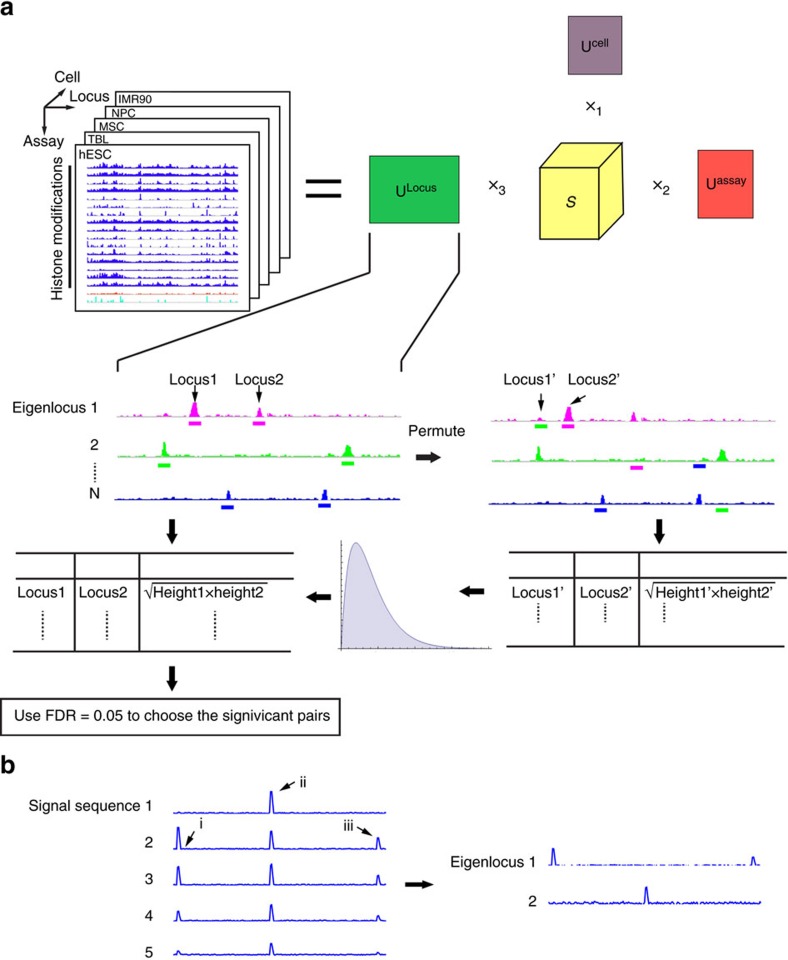
The EpiTensor method. (**a**) An overview of the EpiTensor method. EpiTensor models the epigenomic data with 18 assays in five cell types as a third-order tensor in this study. The three dimensions of the tensor are cell type, assay and genomic locus. EpiTensor uses tensor decomposition technique to decompose the tensor into three subspaces: cell type subspace, assay subspace and locus subspace. The genomic locus subspace involves a set of eigenlocus vectors; each encoding an epigenetic pattern among distal genomic loci. Peaks of eigenlocus vectors were called by MACS2 (ref. 30). Strength of association between two peaks was scored as 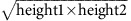, where height1 and height2 are the signal strengths of two peaks, respectively. Eigenlocus peaks were then permuted across eigenloci and the strength of association between two permuted peaks were scored as 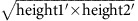, where height1′ and height2′ are the signal strengths of two permuted peaks, respectively. Significant pairs were selected (false discovery rate (FDR)=0.05) on the basis of the distribution of interaction scores between the permuted pairs. (**b**) A simple example to illustrate how PCA extracts association patterns across distal loci. The input are histone mark signals in five cell types and each cell type has three peaks at loci i, ii, and iii (left). The output of PCA includes two eigenlocus vectors, each capturing one spatial association pattern (right).

Genome-wide epigenomes have been mapped with multiple assays in diverse cell types. For a single assay in one cell type, one can represent the genome-wide signal as a vector. For multiple assays in one cell type, one can use a matrix to represent the data. For multiple assays in multiple cell types, a tensor object is required to store the multidimensional nature of the data. Mathematically, a tensor is a higher-order generalization of a matrix. Tensor decomposition is capable of extracting meaningful co-variation patterns from high-dimensional signals^15^,^16^,^17^,^18^. For example, application of tensor decomposition to electroencephalogram (EEG) signals reveals temporal, spectral and spatial patterns of signals from high-dimensional EEG signals^16^. For another example, tensor modelling of face images extracts eigenvectors corresponding to variations of face images under different view, expression and illumination conditions^17^. Here, we used a third-order tensor D_*mnk*_ to model multi-dimensional epigenomic data, where *m*, *n*, *k* are the indices of cell types, assays and genomic loci, respectively (Fig. 1a).

### EpiTensor captures spatial associations between distal loci

To deconvolute epigenomic patterns in the three dimensions, we used higher-order singular value decomposition to decompose the tensor into three subspaces


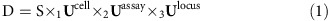


where **U**^cell^, **U**^assay^ and **U**^locus^ are respectively the cell, assay and genomic locus subspace, and S is the core tensor that encodes the interactions among these three subspaces (Fig. 1a, see Supplementary Materials for mathematical details). The three subspace matrices encode the association across cell types, assays and genomic loci, respectively.

Spatial correlation patterns can be derived from analysing eigenvectors in the genomic locus subspace. To illustrate this idea, a simple example of principal component analysis (PCA) is shown in Fig. 1b. Suppose we observe the signals of a single chromatin mark in five cell types at a specific genomic region. Each cell type has three signal peaks and the peak heights vary across cell types due to the cell-specificity of histone modifications. Obviously, peaks i and iii co-vary across the five cell types although they are separated by peak ii. This spatial association was captured by PCA: principal component 1 (PC1) corresponds to the co-variation of peaks i and iii, and PC2 corresponds to the independent variation of peak ii.

Tensor decomposition captures the patterns of high-dimensional epigenomic data in a similar fashion. Specifically, eigenvectors in the genomic locus subspace **U**^locus^ represent spatial association patterns and peaks on the eigenvectors correspond to the associated regions (Fig. 1a). In this study, we analysed 16 histone modifications (H2BK12ac, H3K14ac, H3K18ac, H3K23ac, H3K27ac, H3K27me3, H3K36me3, H3K4ac, H3K4me1, H3K4me2, H3K4me3, H3K79me1, H3K9ac, H3K9me3, H4K8ac and H3K91ac), DNaseI-seq and RNA-seq data in five cell types including human embryonic stem cells (hESCs), TBL cells, MSCs, NPCs and human lung fibroblasts (IMR90) cells. To accelerate computation, we perform our analysis in TADs because previous studies showed that physical interactions mainly occur within TADs^5^,^21^,^22^.

**Figure 2:**
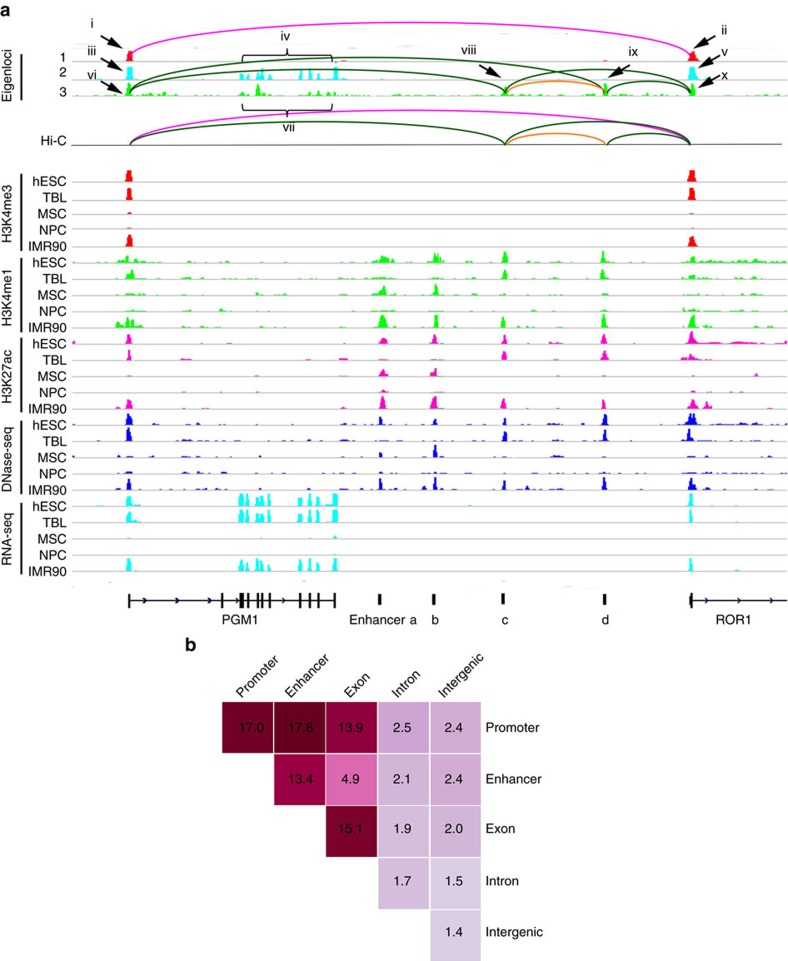
EpiTensor captures spatial patterns across distal genomic loci. (**a**) An example of locus subspace from the EpiTensor analysis Peaks i-ix are peaks in eigenloci 1-3. PGM1 and ROR1 promoters as well as enhancers (marked as enhancers a-d) are annotated in the bottom track (see text for details). (**b**) The distribution of eigenlocus peak pairs (percentage) at promoters, enhancers, exons, introns and intergenic regions.

An example of spatial association captured by EpiTensor is shown in Fig. 2a. Peaks i and ii in the first eigenlocus vector overlap with H3K4me3 peaks at PGM1 and ROR1 promoters. Obviously, H3K4me3 has similar activity profiles in these two promoters: high levels in hESC, trophoblast-like (TBL) and IMR90 cells, and low signals in mesenchymal stem cells (MSCs) and neural progenitor cells (NPCs). This H3K4me3 co-enrichment pattern is captured by the first eigenlocus vector. The second eigenlocus vector has peaks iii, iv and v, coincident with the co-expression of RNA-seq signals at PGM1 and ROR1 exons. The third eigenlocus vector has peaks vi–x: peaks vi and x overlap with PGM1 and ROR1 promoters; peaks viii and ix are enhancers previously predicted by a computational method called RFECS using chromatin signatures in these five cell types (see Supplementary Methods)^19^,^20^. Notably, the H3K4me3 profiles at PGM1 and ROR1 promoters are highly correlated with the H3K4me1 and H3K27ac profiles at enhancers c and d. This coordinated activity profiles between promoter and enhancer is captured by the third eigenlocus. There are another two enhancers (enhancers a and b) in the neighbouring regions. However, their activity profiles (H3K4me1 and H3K27ac) are not correlated with the H3K4me3 profiles at PGM1 and ROR1 promoters, and thus are not captured by the third eigenlocus vector. An additional example of capturing distal associations by eigenlocus vectors is shown in Supplementary Fig. 1.

Previous computational methods have largely focused on predicting promoter–enhancer interactions using supervised learning methods^13^,^14^ or correlation between promoter and enhancer activity profiles^23^,^24^,^25^,^26^,^27^,^28^,^29^. Supervised learning methods are limited by the uncertainty associated with the labels used for training. In practice, correlation-based methods consider only a subset of chromatin modifications and thus cannot capture complex epigenomic patterns in the genome. In contrast, EpiTensor is an unsupervised method (without using prior knowledge), in which promoter–enhancer interactions are discovered *de novo* and thus is independent of Hi-C and ChIA-PET assays. EpiTensor is also equipped with the capability to deconvolute complex covariation patterns in high-dimensional space. Furthermore, previous computational methods largely focused on promoter–enhancer interactions while EpiTensor discovers other types of interactions, such as promoter–promoter and enhancer–enhancer interactions.

### Characterization of spatial associations

To identify spatially associated pairs, we repeated the following four steps for each eigenlocus: (1) We called peaks in each eigenlocus using MACS2 software package^30^ with default parameters; (2) In each TAD, we defined a spatial association score 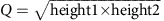 for each pair of peaks on one eigenlocus, where height1 and height2 are the strength of two peaks, respectively; (3) We permuted peaks to form random pairs and computed randomized spatial association score *Q*_random_ using the same definition above; and (4) Peak pairs were chosen with a false discovery rate ≤0.05 based on the *Q*_random_ distribution.

To characterize the spatial associations identified by EpiTensor, we categorized them into groups that contain promoters, enhancers, exons, introns and intergenic regions (Fig. 2b). Promoters and introns were defined by combining RefSeq genes with ‘NR' and GENCODE 19 noncoding genes. Enhancers were predicted in the previous studies using the Random Forest for Enhancer Identification using Chromatin State (RFECS) method^19^. Intergenic regions were defined as the remaining portion of the genome not overlapping with annotated promoters, enhancers, exons and introns (see ‘Methods' section for detailed description of genome annotation). We identified 500,721 pairs of associations in total (we considered all pairwise associations between loci having eigenlocus peaks). The top five groups are promoter–enhancer (17.8%), promoter–promoter (17.0%), exon–exon (15.1%), promoter–exon (13.9%) and enhancer–enhancer (13.4%). All the other types of associations occupy less than 5% of the total pairs.

**Figure 3:**
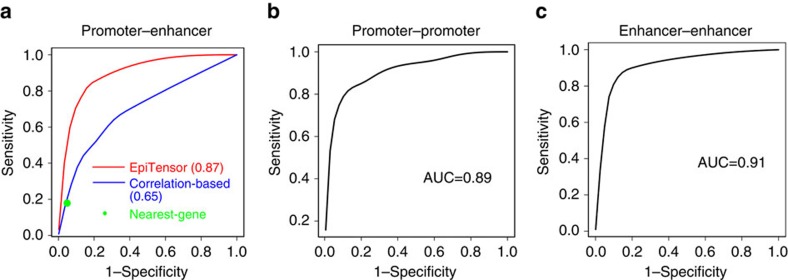
The prediction ROC analysis. (**a**) promoter–promoter associations. (**b**) promoter–enhancer associations. (**c**) enhancer–enhancer associations. The prediction results were compared against the ones from the high-resolution Hi-C data in IMR90. The EpiTensor prediction accuracy of promoter–enhancer interactions was also compared against the ones from correlation-based and nearest gene-based methods.

To examine whether these five groups of associations are resulted from physical interactions, we first compared them with interactions detected by Hi-C experiments. We downloaded the Hi-C data in IMR90 cells from Jin *et al*. study^5^, which has a resolution of 5–10 kb, the highest resolution Hi-C data when this study was conducted. In Jin *et al*. paper^5^, an improved data filtering strategy was used to remove illegitimate interactions based on the strand information of Hi-C paired-end reads. Random collision frequencies between HindIII restriction fragments were modelled by taking into account mappability, fragment size and GC content. This step is in spirit close to the normalization step by Yaffe and Tanay^31^ with some modifications, such as that distance and fragment size were normalized jointly and GC content was treated independently. These modifications allow accurate identification of short-range interaction between chromatins. A negative binomial distribution was then fitted to assess the significance of contact frequency in comparison to random collision between chromatin fragments. This Hi-C data set was previously used to identify promoter–enhancer interactions^5^. We downloaded the identified interactions (see ‘Methods' section and ref. 5 for detailed description of Hi-C data processing) and extracted Hi-C interactions between active promoters and enhancers in IMR90 cells. To compute the area under the curve (AUC), we ranked the association from EpiTensor in terms of their association scores. True positives were defined as EpiTensor predictions validated by Hi-C experiments, false positives as predictions not validated by Hi-C experiments, false negatives as interactions not predicted by EpiTensor but found by Hi- C experiments and true negatives as interactions not predicted and not found by Hi-C experiments. By gradually changing the association threshold, a series of sensitivity and specificity values were computed and these values were used to plot the receiver operating characteristic (ROC) curve. The area under the ROC curve was computed accordingly. Comparison between EpiTensor and Hi-C interactions on these active promoters and enhancers in IMR90 cells gave an impressive AUC of the ROC curve of 0.87. Similar analyses showed that AUCs are 0.89 and 0.91 for promoter–promoter and enhancer–enhancers associations, respectively (Fig. 3b and c). These results indicate that promoter–promoter, promoter–enhancer and enhancer–enhancer associations are largely due to physical contacts. It should be noted that no inter-TAD interactions from Hi-C data were removed in the comparison, indicating that majority of the physical interactions occurred within TADs. This is consistent with previous observations^5^,^21^,^22^. In contrast, the AUCs for the promoter–exon and exon–exon categories were 0.51 and 0.57, respectively (Supplementary Fig. 3a and b); the associations in these two categories are not related to physical interactions (Supplementary Fig. 2).

To further illuminate the success of EpiTensor in identifying the spatial associations resulted from physical interactions, we compared its performance on predicting promoter–enhancer interactions with two other commonly used methods: nearest-gene assignment and a correlation-based method^23^. The nearest-gene assignment approach assigns enhancer to its nearest active promoter. The correlation-based method is based on the multi-cell-type correlation between gene expression levels and histone modifications associated with enhancer activity (H3K4me1, H3K4me2 and H3K27ac), as described in ref. 23. Both of these methods performed much worse than EpiTensor (AUC=0.87 for EpiTensor versus AUC=0.65 for correlation-based method; Fig. 3a).

Next, we compared EpiTensor prediction with another set of high-resolution Hi-C data reported in ref. 6. This set of data include 5 kbp-resolution Hi-C data in GM12878, HMEC, HUVEC, IMR90, K562 and NHEK cells. We repeated the above comparison analysis and obtained an AUC of 0.76–0.89 for promoter–promoter interactions, 0.73–0.87 for promoter–enhancer interactions and 0.74–0.89 for enhancer–enhancer interactions (Supplementary Figure 5). It should be noted that the number of Hi-C interactions is smaller in ref. 6 than that in ref. 5. When computing the AUC, one varies association strength threshold at multiple confidence levels when comparing with Hi-C interactions. EpiTensor achieves high AUCs when comparing with both data sets, indicating that it is consistent with Hi-C interactions from ref. 6 at a higher confidence level while consistent with Hi-C interactions from ref. 5 at a lower confidence level.

To further validate with other types of 3D interaction data, we compared EpiTensor prediction with ChIA-PET data. As there is no ChIA-PET experiment available in any of the cell types that we have analysed using EpiTensor, we chose to use ChIA-PET data in K562 cells for comparison. We extracted pairs between active promoters/enhancers in K562 cells and compared them with interactions of active promoters detected by ChIA-PET experiments (see Supplementary Note 1. The AUCs are 0.81, 0.86 and 0.76 for promoter–promoter, promoter–enhancer and enhancer–enhancer interactions (Supplementary Fig. 5a–c). Furthermore, we assessed the EpiTensor performance using expression Quantitative Trait Loci (eQTL) data in HepG2 (ref. 32) and GM12878 cells^33^,^34^,^35^ (see Supplementary Note 1) and computed the percentage of eQTLs predicted by EpiTensor. Remarkably, EpiTensor predicted 66.7% and 44.9% of eQTL determined by promoter–enhancer interactions in these liver and lymphoblast cell lines, respectively (Supplementary Fig. 5d), which are significantly higher (*P* value <10^−14^ by binomial test) than those from random pairs (7.4% and 11.1% for liver and lymphoblast cell lines, respectively). Promoter–enhancer interactions from EpiTensor were significantly enriched for SNPs correlated with gene expression levels (*P* value <10^−14^ by binomial test, Supplementary Fig. 5d).

Taken together, these statistics confirmed that the spatial interactions are successfully captured from the original multi-dimensional epigenomic signals by EpiTensor. Note that EpiTensor identifies these spatial interactions without using any prior knowledge. Rather, it *de novo* derives 3D interactions from 1D epigenomic assays.

### Validation of predicted interactions with 3C experiments

**Figure 4:**
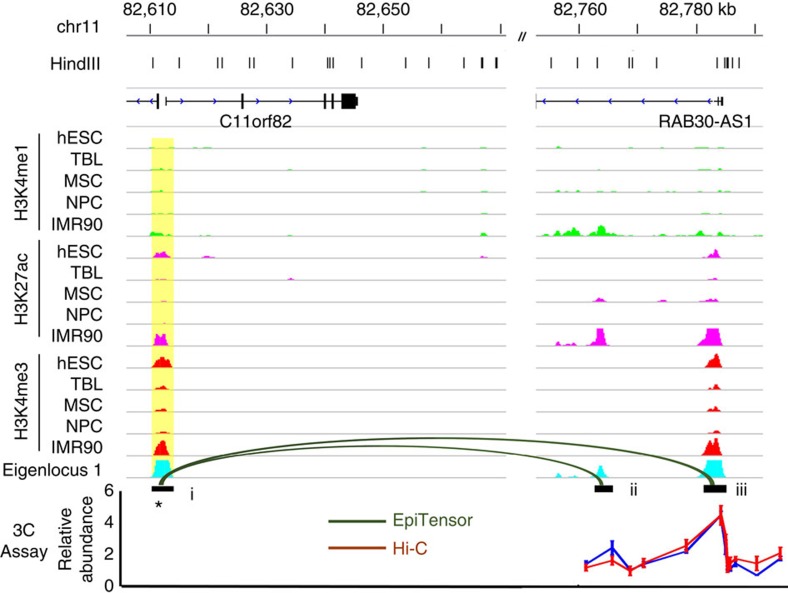
3C Validation of two distal interactions identified in IMR90 cells. These two interactions are between C11orf82 promoter and RAB30-AS1 promoter (pair i and iii), and between C11orf82 promoter and a predicted enhancer (pair i and ii). Anchor fragment chosen in 3C is marked with an asterisk and highlighted in yellow. These two interactions were not identified by Hi-C experiments in IMR90 cells. The red and blue lines in 3C signal panel represent two biological replicates. Each biological replicate is averaged from three technical replicates.

To further assess the performance of EpiTensor, we performed chromosome conformation capture coupled with quantitative PCR (3C-qPCR) on 14 randomly selected pairs from EpiTensor. We achieved a 93% validation rate (13 out of 14), compared with a detection rate of 50% by Hi-C (7 out of 14; Fig. 4 and Supplementary Fig. 7). As shown in Fig. 4, eigenvector 1 from tensor decomposition has three peaks, that is, loci i, ii and iii. Loci i and iii correspond to the C11orf82 and RAB30-AS1 promoters, respectively, while locus ii corresponds to an active enhancer in IMR90 with H3K4me1/H3K27ac enrichment. Locus i was used as anchor and loci ii and ii were used as test sites. Two 3C signal enrichments were observed at loci ii and iii, respectively. The 3C signal peak at locus iii corresponds to the interaction between C11orf82 and RAB30-AS1 promoters. Strong correlation of H3K4me3 signals between these two promoters were observed because both were enriched with H3K4me3 in hESC and IMR90 cells but not in TBL, MSC and NPC cells. This strong correlation was captured by eigenlocus 1 with two peaks at loci i and iii, respectively. The second pair of interaction was between loci i and ii. Locus ii has strong H3K4me1/H3K27ac signals in IMR90 cells, but not in the other four cell types, leading to a small peak at locus ii in eigenlocus 1; this peak results from a combination of multiple signals by EpiTensor, an advantage of EpiTensor compared with correlation-based methods. This small peak at locus ii corresponds to a weaker 3C signal peak in comparison with locus iii. More 3C validation results are shown in Supplementary Fig. 7. It should be noted that five validated interactions were over 300 kb long, two of which were not detected by the Hi-C experiment in IMR90 cells, indicating that EpiTensor can accurately predict long-range interactions.

### Interaction hotspots

We observe that associations are not uniformly distributed among promoters and enhancers, consistent with previous studies that some loci are involved in many interactions^36^. Here, we selected the top 10% promoters and enhancers with the highest interactions degrees (>6 interactions for promoters in promoter–promoter interactions, >5 interactions for promoters and >7 interactions for enhancers in promoter–enhancer interactions and >4 interactions in enhancer–enhancer interactions) and dubbed them as interaction ‘hotspots.' Altogether, we identified 2,673 promoter hotpots in promoter–promoter interactions, 3,702 promoter and 3,875 enhancer hotspots in promoter–enhancer interactions and 5,800 enhancer hotspots in enhancer–enhancer interactions.

To understand the biological implication of the 3D interactions found by EpiTensor, we examined multiple genomic and epigenomic signals and found that hotspots are characterized by distinct features.

First, hotspots have higher chromatin activity across cell types. We initially compared the six core histone marks (H3K4me1, H3K4me3, H3K27ac, H3K27me3, H3K36me3 and H3K9me3) and DNaseI-seq profiles between hotspots and non-hotspots in each of the five cell types (non-hotspots are promoters/enhancers other than hotspots). Hotspots showed slightly stronger enrichment of H3K4me1, H3K4me3, H3K27ac and DNaseI-seq in comparison with non-hotspots (Supplementary Figs 8–11), suggesting that hotspots and non-hotspots have similar levels of chromatin activity in an individual cell type.

**Figure 5:**
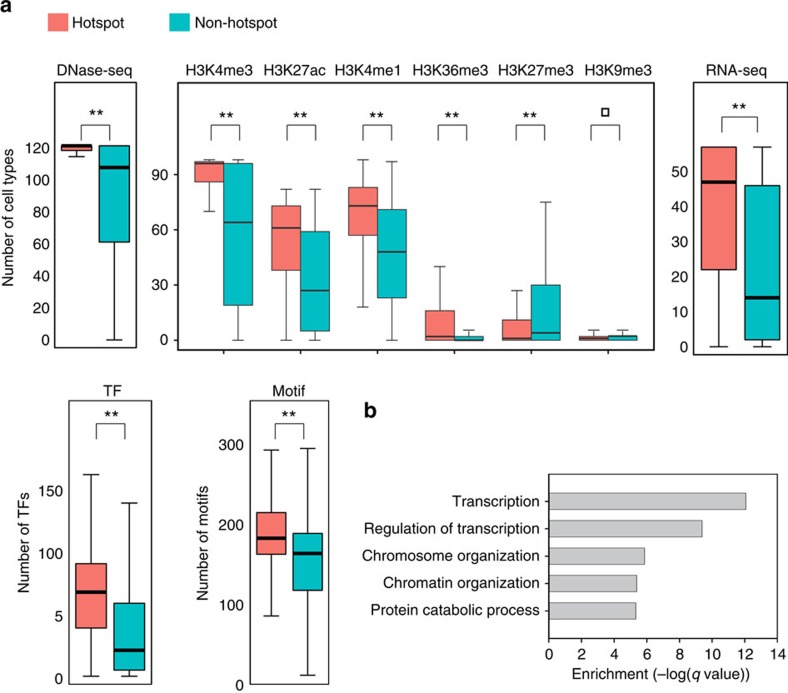
Characterization of interaction hotspots in promoter–promoter interactions. (**a**) Comparison of hotspots and non-hotspots in terms of chromatin accessibility, histone modifications, gene expression, TF binding and motif enrichment. Peaks of DNaseI-seq, histone modification and TF ChIP-seq data in around 100 cell types (see Supplementary Tables 1–3 for a complete list) were called and the occurrence frequency of peaks were counted for each hotspot and non-hotspot promoter. RPKM values from RNA-seq data in 57 cell types (see Supplementary Table 1 for a complete list) were used to classify promoters into expressed and non-expressed ones (RPKM cut-off=5) and occurrence frequency of expressed promoters were counted for each hotspot and non-hotpot promoter. Motif enrichment values were used to classify each of the 292 TFs as being present or absent in each hotspot and non-hotspot promoter (motif enrichment cut-off=0.6, see ‘Methods' section). Occurrence frequency of present motifs were counted for each hotspot and non-hotspot promoter. *P* values calculated using Wilcoxon test are denoted as **P≤0.001; ^□^*P*>0.05. (**b**) GO term analysis of hotspot promoters.

However, much more significant difference was observed when we pooled together the DNaseI-seq and the six core histone mark ChIP-seq data in 82–125 cell types; including cell lines, primary cells and tissue (see Supplementary Tables 1 and 2). We overlapped their peaks with each hotspot/non-hotspot and counted the number of cell types with overlapping peaks. Obviously, hotspots are more tightly associated with active histone marks H3K4me1, H3K4me3, H3K27ac and DNaseI-seq peaks across cell types (Fig. 5a and Supplementary Figs 12–14, *P* value <2.2 × 10^−16^, Wilcoxon test). These results indicate that hotpots have higher chromatin activity than non-hotspots across cell types.

Second, hotspot promoters are associated with highly expressed genes across cell types. We collected Reads Per Kilobase of transcript per Million mapped reads (RPKM) values from RNA-seq data in 57 cell types (see Supplementary Table 1) and counted the number of cell types with expressed genes for each hotspot and non-hotspot. Hotspot promoters have significantly higher number of cell types with expressed genes than non-hotspots (Fig. 5a, *P* value <2.2 × 10^−16^, Wilcoxon test).

Third, hotspots are enriched for TF binding sites. We collected the ChIP-seq data for 49, 98 and 77 TFs mapped by the ENCODE consortium^28^ in H1, K562 and GM12878 cells, respectively (see Supplementary Table 3). We counted the ChIP-seq peaks in each hotspot and compared the occurrence frequency with that in non-hotpots. Hotspots have a higher TF binding preference than non-hotspots (Fig. 5a, *P* value <2.2 × 10^−16^, Wilcoxon test). Previous studies have shown that TF bindings are not uniformly distributed but occupy specific loci referred as high occurrence target regions^37^,^38^,^39^,^40^. It is well known that DNA binding factors such as CTCF and cohesin can stabilize chromatin structures^41^,^42^,^43^. Our analysis suggests that the formation of clustered TF binding is related to 3D chromatin structure.

These hotspots are also enriched for TF motifs. As ChIP-seq data are available only for a limited number of TFs, we collected a comprehensive set of sequence motifs known to be recognized by TFs and other DNA binding proteins from five databases (see ‘Methods' section). We computed the score of each motif in hotspots and non-hotspots. Figure 5a shows the significant enrichment of motif scores in the hotspots. This observation is unexpected but consistent with the enriched TF ChIP-seq peaks across cell types, which indicates these hotspots having specific sequence features.

Fourth, hotspots have significant overlap with lncRNA binding sites. As lncRNAs have been shown to be important for chromatin structure organization^44^,^45^,^46^, we examined the binding sites of two human lncRNAs, *NEAT1* and *MALAT1*. Both *NEAT1* and *MALAT1* were shown to bind to active chromatin sites^47^. Moreover, *MALAT1* localizes to nuclear speckles (interchromatin nuclear domains enriched for serine/arginine splicing factors)^48^ and *NEAT1* is required for formation of paraspeckles (nuclear bodies close to nuclear speckles)^49^. Although the binding sites of these two lncRNAs were determined in MCF-7 cells, not in any of the five cell types used to build the EpiTensor model, their binding sites have significant overlap with the hotspots (*P* value <9.2 × 10^−5^, hypergeometric test). This significant difference suggests preferred binding of these two lncRNAs to the hotspots. Both *NEAT1* and *MALAT1* are important for transcription and the enrichment of their binding sites in hotspots is consistent with the observation that hotspots are associated with active chromatin structure across cell types.

Fifth, hotspots have significant overlap with super enhancers. We collected super enhancers in 96 human cell types/tissues. We overlapped them with each hotspot/non-hotspot and counted the number of cell types with overlapping super enhancers (Supplementary Figs 13 and 14). Our results showed that hotspots are more likely to overlap with super enhancers than non-hotspots.

Last, hotspots are involved in important biological functions. In the promoter–promoter association category, the hotspot promoters are enriched with ‘chromatin organization' and ‘chromosome organization' (Fig. 5b), suggesting that these interactions mediated by the hotspot promoters are crucial for the formation and maintenance of proper chromatin structure that allows precise promoter communication and gene regulation. For example, the gene SMC1A, which belongs to the structural maintenance of chromosome (SMC) family, is involved in chromosome cohesion during cell cycle and DNA repair^50^ and a hotspot is found at its promoter. SMC1 forms a cohesion complex with SMC3, another gene in the SMC family, to hold sister chromatids together for correct segregation of chromosomes during cell division^51^. This process requires ATP hydrolysis for the stable association of cohesion with chromosomes^52^. Mutation of SMC1A gene abolished ATP hydrolysis, leading to the inhibition of loading of the complex to chromosomes and failure of chromosome cohesion^52^. For another example, SMARCA1 and BPTF genes are the components of the Nucleosome Remodeling Factor, which is crucial for chromatin remodelling, nucleosome rearrangement and high-order chromatin structure formation^53^,^54^. In the promoter–enhancer category, hotspot enhancers are enriched with ‘regulation of cell shape' and ‘immune system functions' (Supplementary Fig. 13b). This indicates the importance of these enhancers in the complex regulation of immune system in response to cellular and environmental conditions, consistent with the observations in ref. 25, which shows that complex regulated genes are markedly enriched for immune system functions. The hotspot promoters in this category are related to important cell physiology functions, including metabolic process and cell motion (Supplementary Fig. 12b). The hotspot enhancers in enhancer–enhancer association category play roles in cell adhesion and intracellular transport (Supplementary Fig. 14b), indicating the importance of genes to control the interactions of cells with their niche and signalling environments.

In summary, interaction hotspots are associated with higher chromatin and transcriptional activity across cell types. They are preferred for TF binding and are enriched with TF motifs. Furthermore, these loci are also preferably bound by lncRNAs. Interaction hotspots are linked to multiple partners and provide a topological framework for coordinated transcription or regulation of the associated regions.

## Discussion

Our study presents the first attempt to deduce 3D spatial epigenomic patterns from 1D assays that provides a new method complementary to Hi-C and ChIA-PET. EpiTensor decodes the complex co-variation patterns of epigenomic patterns across cell types and genomic locations, which paves the way towards directly linking epigenomic state and chromosomal topology. Such co-variation relationships have previously been used to identify spatial associations but limited to promoter–enhancer interactions using specific marks^23^,^24^,^25^,^26^,^27^,^28^,^29^. In contrast, EpiTensor considers combinatorial effects of diverse epigenomic features, deconvolutes complex covaration patterns in high-dimensional space and identifies many types of associations. We have demonstrated that the promoter–promoter, promoter–enhancer and enhancer–enhancer associations from EpiTensor are highly concordant with those obtained from Hi-C, ChIA-PET and eQTL data.

Complementary to the Hi-C assays that detect physical contacts with a resolution of about 20,000–50,000 bp, EpiTensor analysis can be easily performed at a 200 bp resolution that is sufficient to pinpoint regulatory elements and their associated chromatin states (Supplementary Fig. 4). Constrained by the sequencing cost, Hi-C assays have been performed in limited number of cell types. In contrast, a deluge of epigenomic data has been generated by the NIH Roadmap Epigenomics Project^55^, which can be used to derive 3D interaction maps using EpiTensor. Importantly, EpiTensor analysis considers chromatin state tightly associated with transcriptional activity and it thus provides a view of chromosomal organization orthogonal to Hi-C that only detects physical contacts.

Previous studies have shown that topological structures may exist before the modification of histones; these interactions can be captured by EpiTensor. In the integrated analysis across cell types, as long as a locus is marked by a variation in epigenetic modifications in some cell types, its interactions with other loci can be detected by EpiTensor. For example, physical contacts can exist before enhancers become active and such interactions can be naturally captured by EpiTensor because the poised or inactive enhancers are marked by H3K27me3 and this mark is removed when the enhancers become active in other cell types to regulate the target genes. In this study, we focused on identifying interactions between active promoters or enhancers that are critical for transcriptional regulation. An intriguing observation is that the EpiTensor interactions showed high concordance with active promoter–promoter, promoter–enhancer, enhancer–enhancer interactions from Hi-C data of two independent studies in 6 human cell lines and ChIA-PET data in K562 cells, indicating the power of EpiTensor to identify biologically important interactions.

The interaction hotspots of promoters and enhancers are located in genomic regions with significantly higher chromatin and transcriptional activities across cell types. These hotspots are also enriched with TF and lncRNA binding as well as TF motif presence. The spatial interactions of these hotspots are highly concordant with the Hi-C, ChIA-PET and eQTL data, and the biological functions are highly relevant to chromosomal organization. Taken together, these observations indicate that the interaction hotspots identified by EpiTensor are important in linking 3D genome structure to functional activity. It is worth noting that the hotspots have little overlap with the loci that are involved in many 3D contact detected by Hi-C experiments, which is not surprising as the 3D contact loci are buried inside chromosome and the hotspots are functional sites exposed on the surface of chromosome structure. It is tempting to speculate that these hotspots are critical for stabilizing the genomes 3D topology; however, this hypothesis awaits experimental test.

## Methods

### Data

Genome-wide maps of 16 chromatin modifications (H2BK12ac, H3K14ac, H3K18ac, H3K23ac, H3K27ac, H3K27me3, H3K36me3, H3K4ac, H3K4me1, H3K4me2, H3K4me3, H3K79me1, H3K9ac, H3K9me3, H4K8ac and H4K91ac), RNA-seq and DNaseI-seq in hESCs, TBL cells, MSCs, NPCs and human lung fibroblast cells (IMR90) were downloaded from the website of NIH Roadmap Epigenomics project (http://www.roadmapepigenomics.org/). The downloaded data were in BED format. The spp software^56^ was used to compute the tag density profile for each data set. Specifically, (1) the BED files were converted to BAM files that were read by the ‘read.bam.tags' function, (2) the ‘remove.local.tag.anomalies' function was used to remove extremely high tag counts relative to the neighbourhood, (3) the ‘get.smoothed.tag.density' function was used with a window size of 200 bp and smoothing bandwidth of 200 bp to compute genome-wide tag density for each data set. The tag density was further transformed to a logarithmic scale for the following analyses.

### EpiTensor

The EpiTensor model is based on high-order tensor decomposition (see Supplementary Materials for details). Let D_*mnk*_ be a third order tensor, where *m* is the cell type, *n* is the assay index and *k* the genomic locus index. Applying tensor decomposition to D, we obtain D=S × _1_**U**^cell^ × _2_**U**^assay^ × **U**^locus^, where **U**^cell^ is the cell type subspace, **U**^assay^ is the assay subspace, **U**^locus^ is the genomic locus subspace and S is the core tensor that governs the interactions among the three subspaces. In this study, we focused on analysing **U**^locus^, which encodes the spatial association among distal loci. Each eigenlocus vector in **U**^locus^represents one epigenomic pattern. Dimension reduction in tensor decomposition was obtained by computing for D, a best rank-(*R*_1_,*R*_2_,…,*R*_*N*_)approximation^57^ 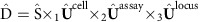, which minimizes the error function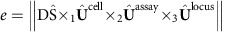, subject to (**U**^cell^)^*T*^ **U**^cell^=I, (**U**^assay^)^*T*^ **U**^assay^=I, and (**U**^locus^)^*T*^ **U**^locus^=I (ref. 58). The three constraints are to ensure orthonormality of the three subspaces. In practice, we used full rank in the cell and assay subspaces because we focus on the locus subspace. In the locus subspace, we chose a rank that keeps at least 95% of the original energy 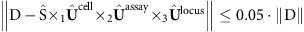.

### Genome annotation

Both coding and noncoding genes were combined to define gene elements. For coding genes, we used the well-curated RefSeq database^59^ and selected the RefSeq IDs starting with ‘NM'. For long noncoding genes, we combined the RefSeq genes starting with ‘NR' and GENCODE 19 long noncoding genes^60^. Promoters, exons and introns were defined according to the combined set of gene annotation. Enhancers were predicted in the previous studies using the Random Forest for Enhancer Identification using Chromatin State (RFECS) method^19^. Briefly, RFECS used histone modification profiles at P300 binding sites to train a Random Forest for enhancer prediction. We further filtered the RFECS-predicted enhancers with H3K27ac peaks to discriminate active enhancers from poised ones^61^. Active enhancers identified in the five cell types were merged to a consensus set of enhancers. Intergenic regions were defined as the remaining portion of the genome not overlapping with annotated promoters, enhancers, exons and introns. We used this genome annotation to divide the spatial associations from EpiTensor into 15 pairwise groups, each involving two types of genome elements.

### Evaluation with Hi-C data

High-resolution Hi-C experiment data were obtained from ref. 5, in which a statistical method was developed to convert raw Hi-C reads into binary interactions by taking into account mappability, fragment size and GC content. A list of anchors covering each HindIII fragment in the genome and the targets of each anchor were downloaded from the supplementary data of ref. 5. We extracted promoter–enhancer pairs between active TSS and active enhancers. The active enhancers were predicted by RFECS^19^ and then filtered by the H3K27ac peaks^20^. The active promoters were defined as TSSs enriched with H3K4me3 but not H3K27me3. These active promoter-active enhancer pairs were compared with those obtained from EpiTensor. The AUC value was computed as the area under the ROC. AUC values are in the range of 0.5–1.0, with 1.0 representing a perfect prediction and 0.5 representing a random prediction. Similar analyses were performed on the other association groups such as promoter–promoter, enhancer–enhancer, promoter–exon and exon–exon associations found by EpiTensor. To further validate the results, another set of high-resolution Hi-C experiment data were obtained from ref. 6 and the same validation comparison was performed.

### Evaluation with ChIA-PET data

ChIA-PET experiment data for H3K4me1, H3K4me2, H3K4me3, H3K27ac, Pol2 and RAD21 in K562 cells were obtained from ref. 22 and merged. Interactions between active promoters and enhancers in K562 cells were extracted from merged ChIA-PET interactions. EpiTensor interactions between active promoters and active enhancers in K562 cells were also extracted. We then performed ROC analysis, as described above, to evaluate the accuracy of promoter–enhancer interactions predicted by EpiTensor. Similar analyses were performed for promoter–promoter and enhancer–enhancer interactions.

### Evaluation with eQTL data

The eQTL data in HepG2 and GM12878 cells were obtained from the University of Chicago QTL browser (http://eqtl.uchicago.edu/cgi-bin/gbrowser/eqtl/). In eQTL evaluation, each SNP that fell within an active enhancer in HepG2/GM12878 cells and was associated with an active gene in HepG2/GM12878 cells was considered eligible for evaluation. We compared the percentage of eQTLs that were predicted by EpiTensor with the percentage from random interaction.

### Validation with 3C-qPCR

3C (chromosome conformation capture) libraries from IMR-90 cells were generated according to standard protocols as described previously in refs 24, 62, 63. In brief, IMR90 cells in exponential growth condition were collected and fixed with 1% formaldehyde, then digested with appropriate restriction enzymes, followed by ligation, reversal of cross-linking and DNA purification to obtain the 3C libraries. The BAC (bacterial artificial chromosome) spanning the regions of interest for validation were used for generating the control libraries that should contain all the possible interactions. The BAC DNA were digested with corresponding restriction enzymes, followed by ligation, reversal of cross-linking and DNA purification to obtain the BAC control libraries. We used real-time qPCR to quantify the ligation frequency. For each interaction site tested, an anchor primer and several test primers were designed to cover the interaction region. The efficiency of each primer sets in qPCR were first corrected with the standard curve obtained from serial dilutions of the corresponding BAC control libraries, then were normalized to the loading of the anchor fragment in each biological replicates. The Ct values of each primer set from the 3C libraries were converted into the relative abundance of PCR products. The relative abundance for each primer set was plotted versus the locations of HindIII fragments to the anchor primer, where a local peak would confirm a specific interaction at this locus. All the qPCR products were verified by Sanger sequencing to confirm the existence of ligation products.

### Characterizing interaction hotspots

The core set of six histone modifications (H3K4me1, H3K4me3, H3K27ac, H3K36me3, H3K27me3 and H3K9me3) were downloaded from NIH Roadmap Epigenomics Project website http://epigenomeatlas.org. As in ref. 64, tight peaks in H3K4me1, H3K4me3 and H3K27ac were called using Homer program ‘findPeaks' with the style ‘histone'^65^. Peaks within 1 kb were merged into a single peak. Broad peaks in H3K36me3, H3K27me3 and H3K9me3 were called using the Homer program ‘findPeaks' with the options ‘-region –size 1000 –minDist 2500'. When Homer runs with these options, the initial sets of peaks were 1 kb wide and peaks within 2.5 kb were merged. Uniform peaks of DNaseI-seq data in 125 cell types were downloaded from the ENCODE website http://genome.ucsc.edu/encode. Uniform peaks of ChIP-seq data for 49, 98 and 77 TFs in hESC, GM12878 and K562 cells, respectively, were downloaded from the ENCODE website.

For histone modification ChIP-seq, DNaseI-seq and TF ChIP-seq data, their peaks were overlapped onto hotspots and non-hotspots, and the number of cell types with overlapping peaks was counted for each hotspot and non-hotspot. Distributions of the number of cell types were compared between hotspots and non-hotspots and Wilcoxon test was used to compute *P* values.

For RNA-seq data, processed RPKM values for RNA-seq data in 57 cell types were downloaded from the NIH Roadmap Epigenomics Project data portal http://epigenomeatlas.org. A cut-off of 5 was used to classify each gene into expressed or un-expressed category in each cell type, and the number of cell types with expressed genes was counted for each hotspot and non-hotspot. Distributions of the number of cell types were compared between hotspots and non-hotspots, and Wilcoxon test was used to compute *P* values.

For motif-enrichment analysis, a library of 292 non-redundant motifs were assembled by combining the DNA binding motifs in Transfac^66^, Jaspar^67^, Uniprobe^68^, hPDI^69^ and Taipale^70^. A PWM scan was performed in each hotspot and non-hotspot region and a motif score was computed. A threshold of 0.6 was used to determine the presence or absence of a motif. The number of motifs present in each hotspot and non-hotspot was counted. Distribution of the number of motifs were compared between hotspots and non-hotspots and Wilcoxon test was used to compute *P* values.

For lncRNA binding enrichment analysis, the binding peaks of *NEAT1* and *MALAT1* were downloaded from ref. 47. We overlapped the binding peaks with each hotspot and non-hotspot and used hypergenomic test to compute the *P* value.

For super enhancer data, we downloaded super enhancers in 96 human cell types/tissues from the dbSUPER website (http://bioinfo.au.tsinghua.edu.cn/dbsuper/index.php). These super enhancers were overlapped onto hotspots and non-hotspots, and the number of cell types with overlapping super enhancers was counted for each hotspot and non-hotspot. Distributions of the number of cell types were compared between hotspots and non-hotpots and Wilcoxon test was used to compare the *P* values.

## Additional information

**How to cite this article**: Zhu, Y. *et al*. Constructing 3D interaction maps from 1D epigenomes. *Nat. Commun.* 7:10812 doi: 10.1038/ncomms10812 (2016).

## Supplementary Material

Supplementary Information Supplementary Figures 1-14, Supplementary Tables 1-4, Supplementary Note 1 and Supplementary References
