# Supplementary material for: Constructing 3D interaction maps from 1D epigenomes
Source: Nat Commun. 2016 Mar 10;7:10812. doi: 10.1038/ncomms10812 (PMC4792925; doi:10.1038/ncomms10812)
Supplement: Supplementary Information — Supplementary Figures 1-14, Supplementary Tables 1-4, Supplementary Note 1 and Supplementary References [file ncomms10812-s1.pdf]

Supplementary Figure 1

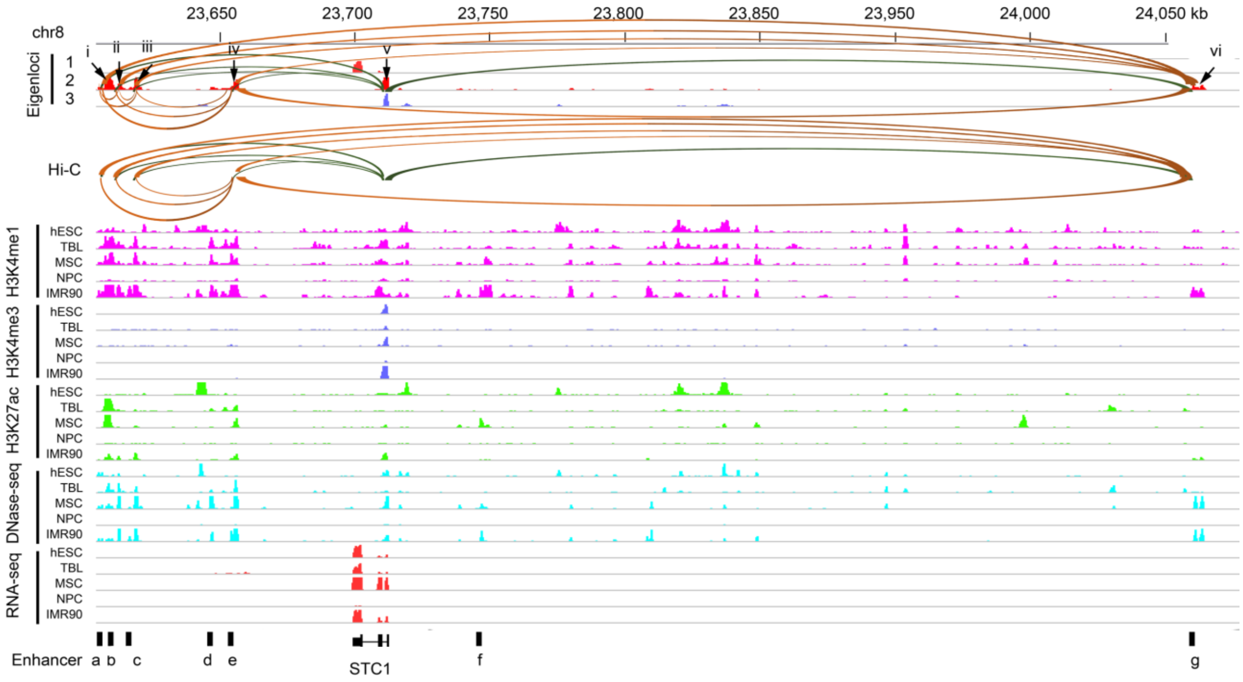

Supplementary Figure 1. An example of eigenloci from Epitensor. The first eigenlocus corresponds to RNA-seq transcripts at STC1. The RNA-seq transcripts have coordinated expression patterns with high levels of signals in hESC, TBL, MSC, and IMR90 cells and low levels of signals in NPC cells. The second eigenlocus involves six peaks, i.e. peak i-vi. Peak i-iv and vi correspond to enhancers a-c and e while peak v corresponds to the promoter of STC1. The third eigenlocus has corresponds to the H3K4me3 activity patterns at promoter STC1. The predicted promoter-enhancer and enhancer-enhancer interactions from EpiTensor are highly concordant from Hi-C interactions.

Supplementary Figure 2

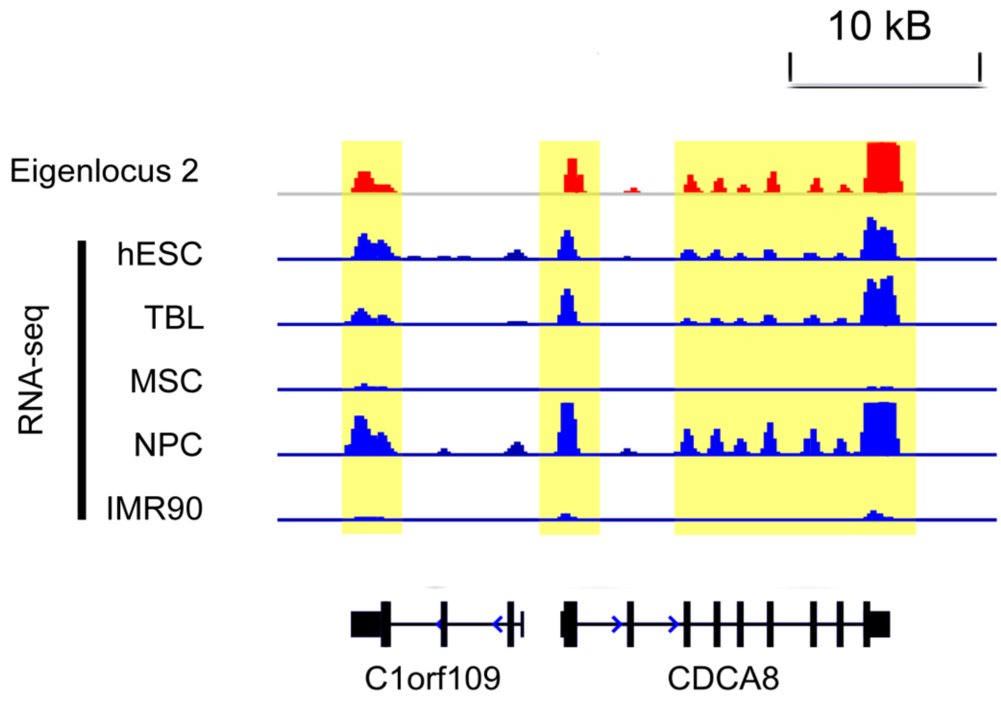

Supplementary Figure 2. An example of exon-exon association identified by EpiTensor.

Supplementary Figure 3

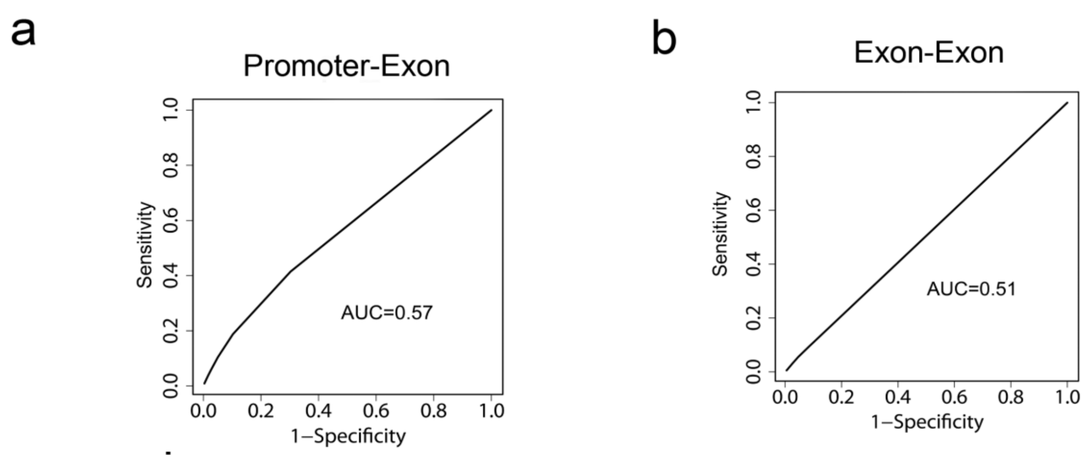

Supplementary Figure 3. The ROC analysis for the prediction of promoter-exon (a), and exon-exon associations (b).

Supplementary Figure 4

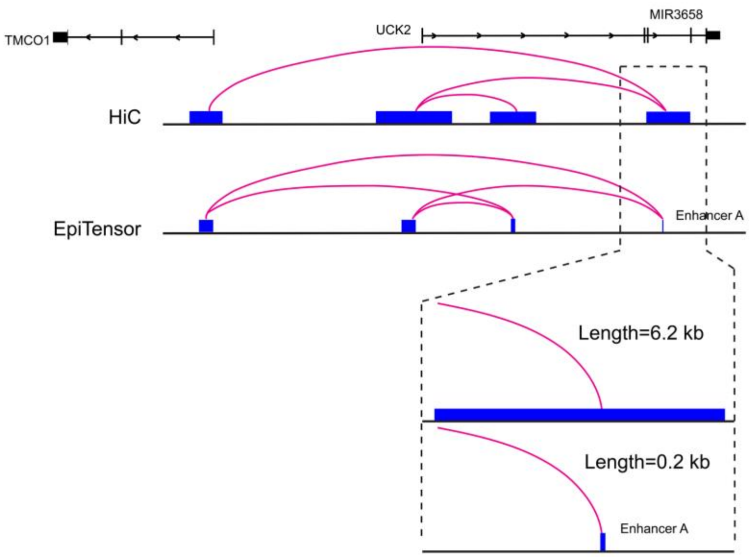

Supplementary Figure 4. Comparison of resolution of Hi-C and EpiTensor interactions. Enhancer A (size=0.2 kb) is more accurately located by EpiTensor than by Hi-C data.

Suppelentary Figure 5

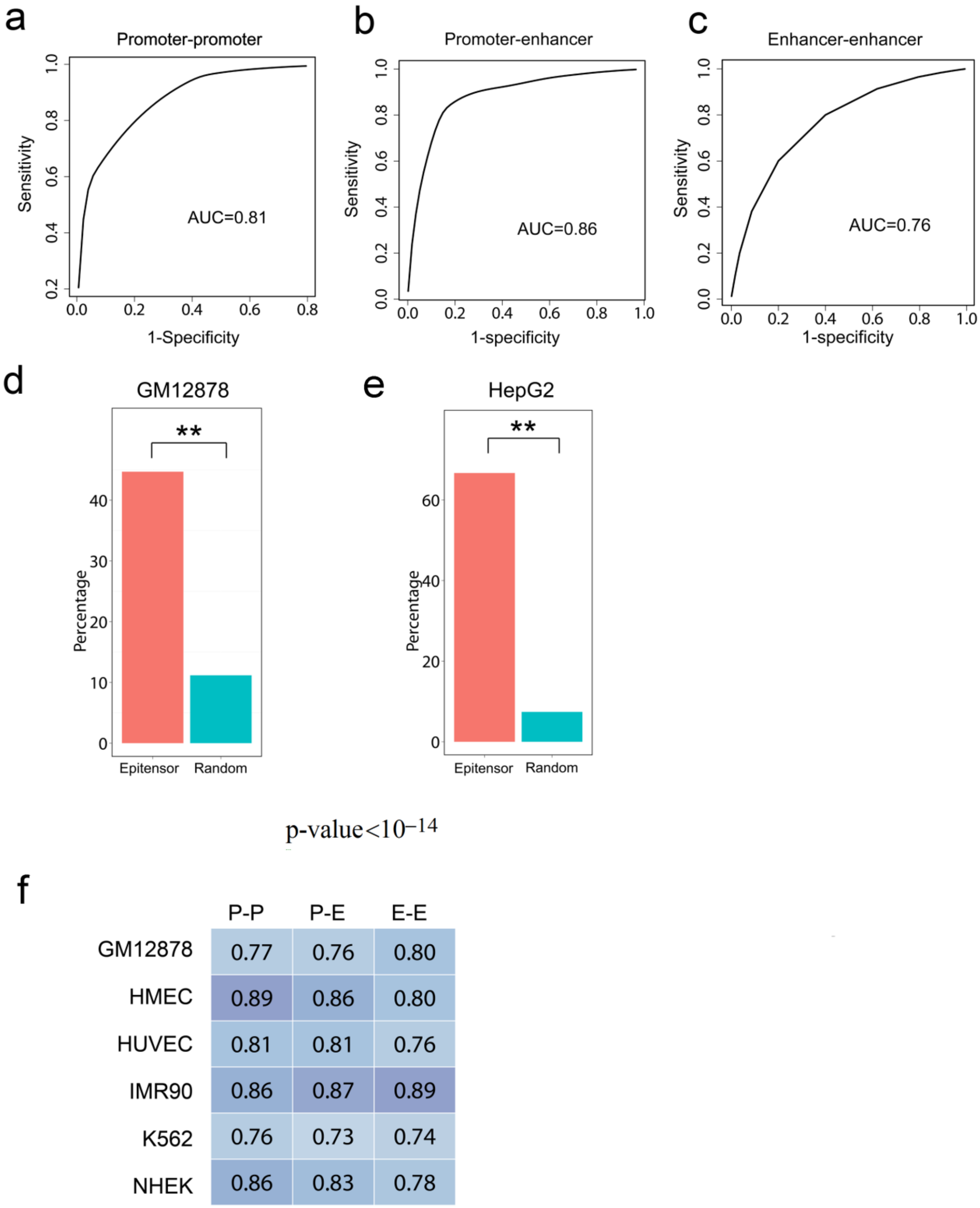

Supplementary Figure 5. (a-c) The ROC analysis for the prediction of promoter-promoter (a), promoter-enhancer (b), and enhancer-enhancer (c) interactions. The prediction results were

compared against the ones from ChIA-PET data in K562 cells. **(d-e)** Validation of EpiTensor prediction with eQTL data in GM12878 (d) and HepG2 (e) cells. Percent of eQTLs predicted by EpiTensor was compared with that from random pairs and p-value was computed using binomial test. **(f)** The AUC values for the prediction of promoter-promoter (P-P), promoter-enhancer (P-E), and enhancer-enhancer (E-E) interactions in GM12878, HMEC, HUVEC, IMR90, K562, and NHEK cells. The predictions results were compared against the ones from high-resolution Hi-C data obtained from Rao et al.

Supplementary Figure 6

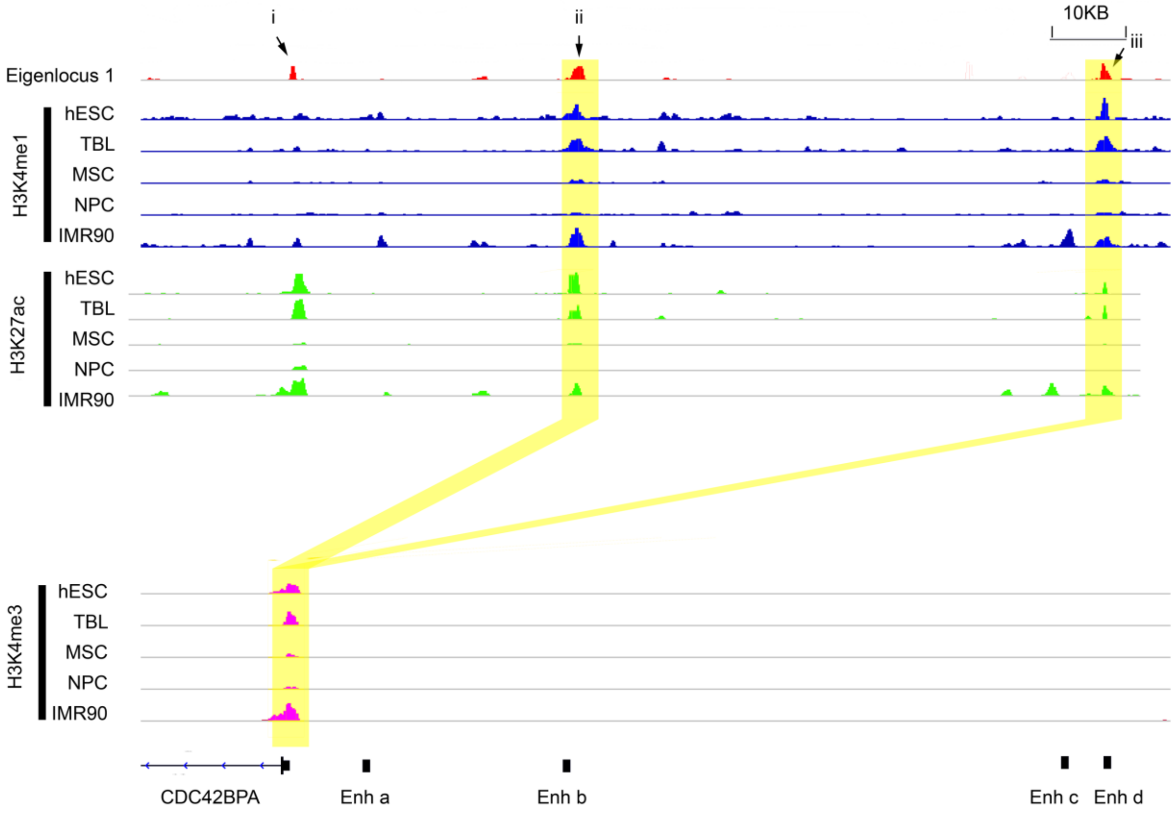

Supplementary Figure 6. An example of promoter-enhancer interaction from EpiTensor. The 1st eigenlocus has three peaks (peaks i, ii, and iii). Peak i falls into the promoter of CDC42BPA while peaks ii and iii overlap with enhancers b and d, respectively. These three peaks co-occur in the same eigenlocus because they have similar epigenetic profiles across five cell types. Specifically, peak i has high levels of H3K4me3 in hESC, TBL, and IMR90 cells, but low levels of H3K4me3 in MSC and NPC cells. Peaks ii and iii has high levels of H3K4me1 and H3K27ac in hESC, TBL, and IMR90 cells but not in MSC and NPC cells. This similar multi-cell epigenetic profile is captured by the 1st eigenlocus vector in the locus subspace. In contrast, enhancer c has high levels of H3K4me1 in IMR90 cells but not in hSC, TBL, MSC, and NPC cells. Enhancer c does not have similar epigenetic profile as CDC42BPA promoter and their peaks do not co-occur in the same eigenlocus vector. Similarly, enhancer a does not appear in the 1st eigenlocus vector either because it has a different epigenetic profile.

Supplementary Figure 7

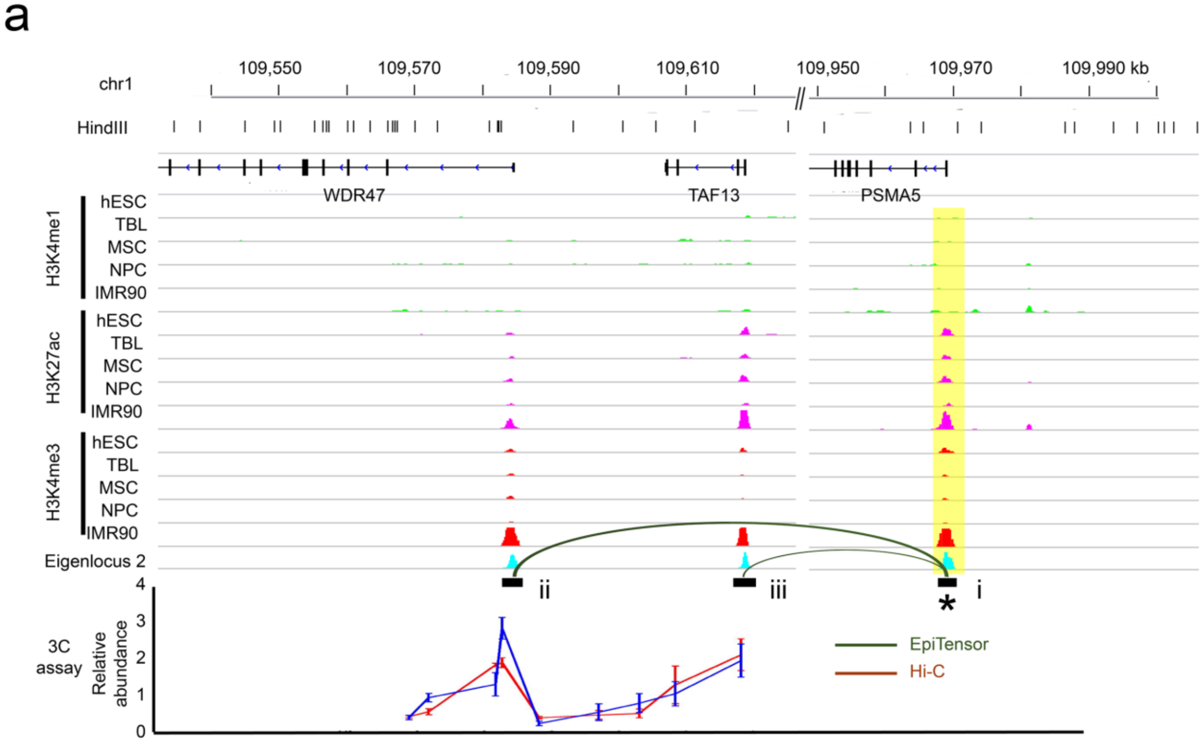

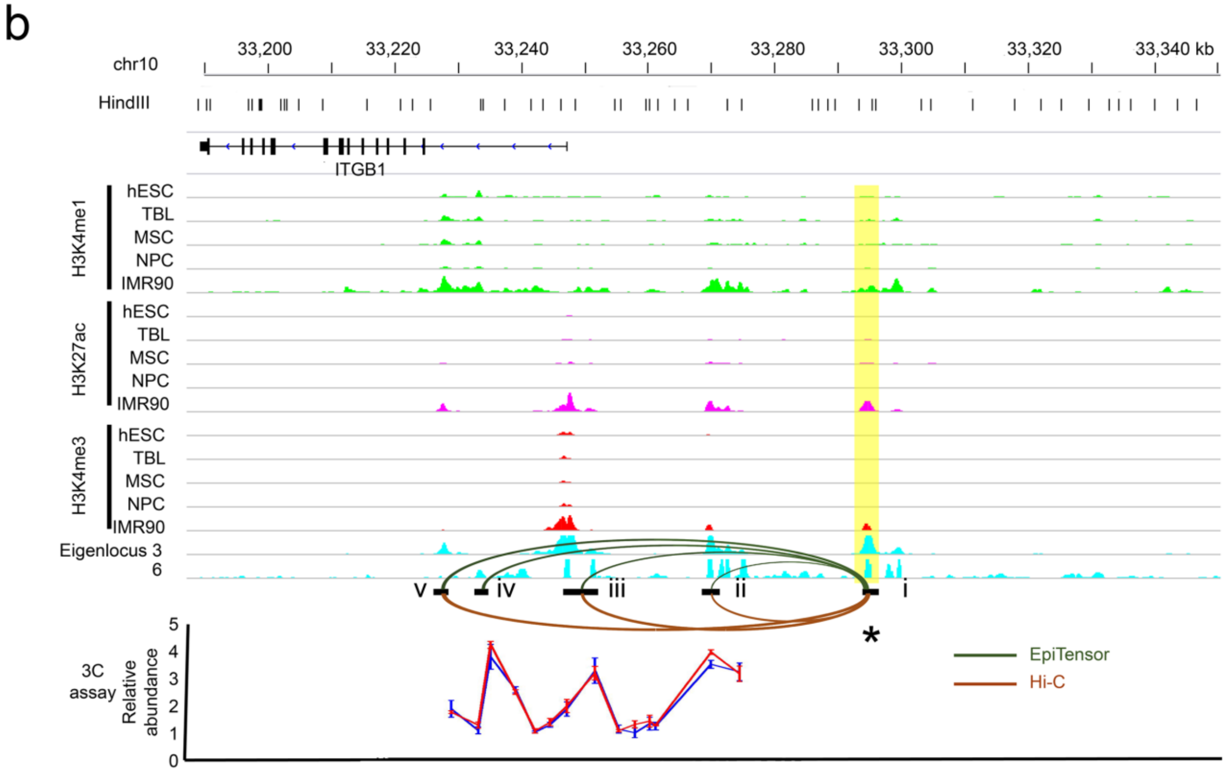

c

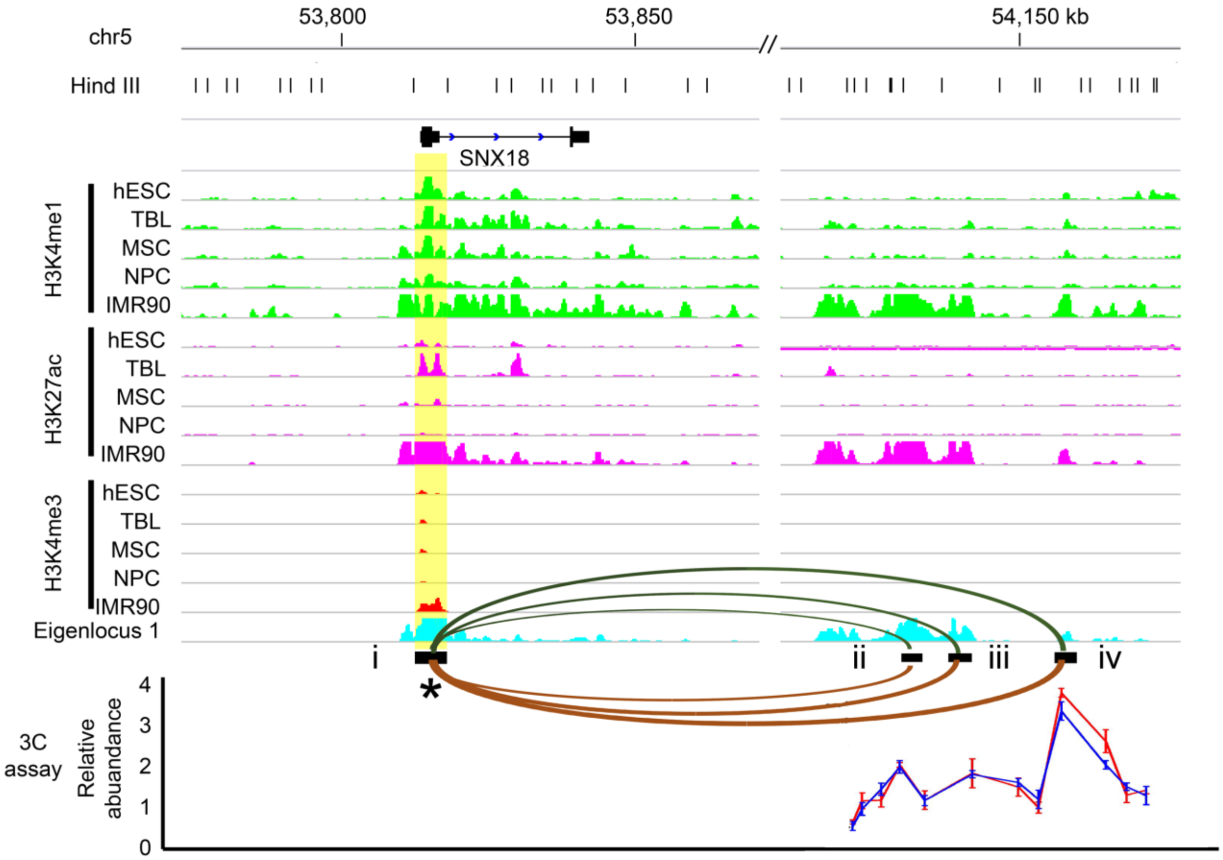
chr5
53,800
53,850
54,150 kb
Hind III
SNX18
H3K4me1
hESC
TBL
MSC
NPC
IMR90
H3K27ac
hESC
TBL
MSC
NPC
IMR90
H3K4me3
hESC
TBL
MSC
NPC
IMR90
Eigenlocus 1
i
ii
iii
iv
*
3C assay
Relative abuandance
4
3
2
1
0

d

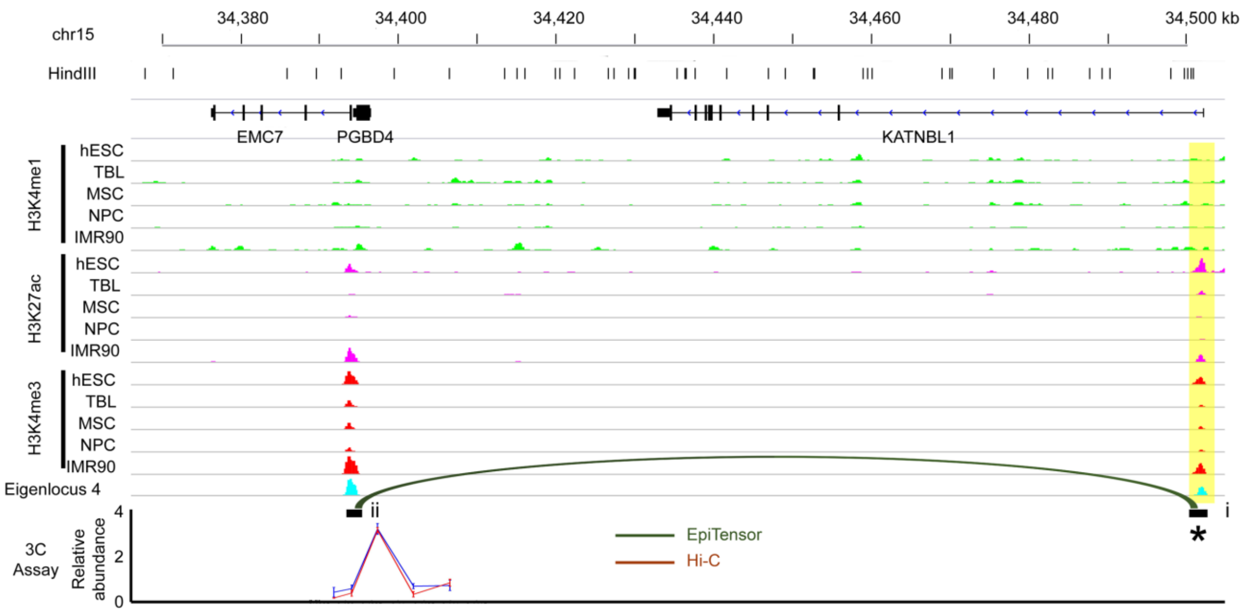
chr15
34,380
34,400
34,420
34,440
34,460
34,480
34,500 kb
HindIII
EMC7
PGBD4
KATNBL1
H3K4me1
hESC
TBL
MSC
NPC
IMR90
H3K27ac
hESC
TBL
MSC
NPC
IMR90
H3K4me3
hESC
TBL
MSC
NPC
IMR90
Eigenlocus 4
ii
i
*
3C Assay
Relative abundance
4
2
0
EpiTensor
Hi-C

e

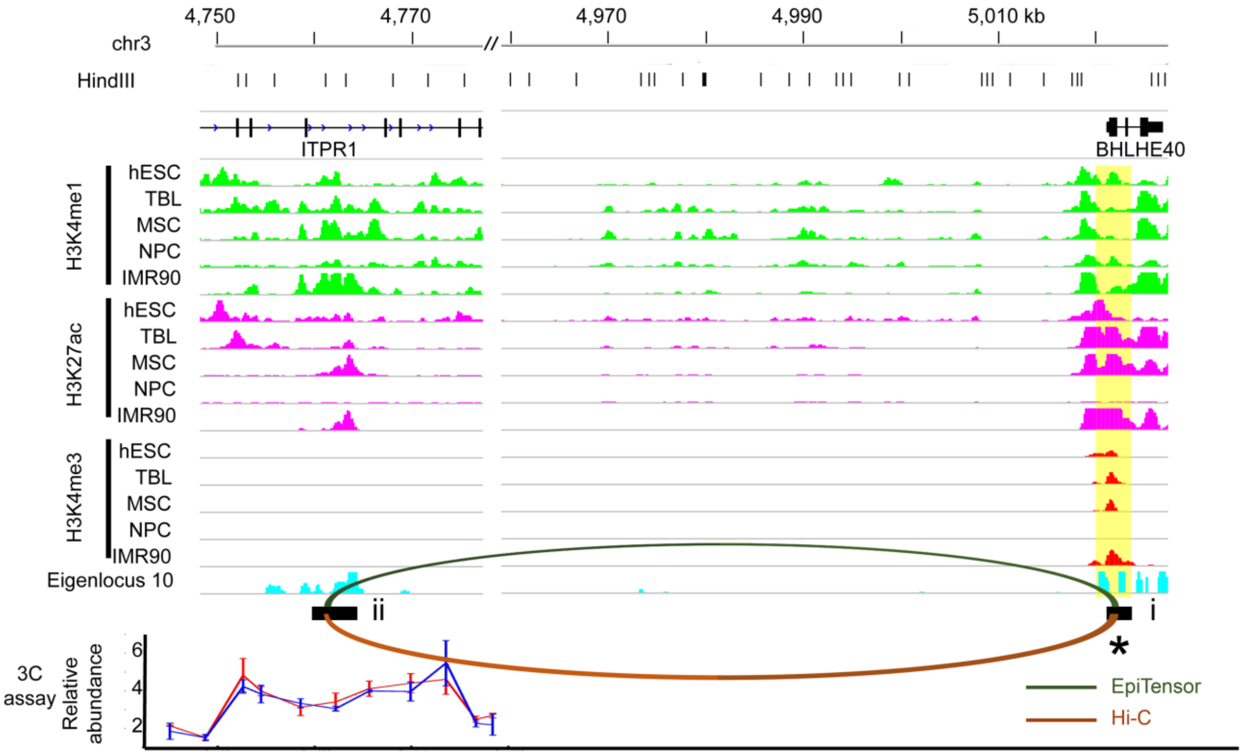

Supplementary Figure 7. 3C Validation of distal interactions identified in IMR90 cells. (**a**) Validation of two distal interactions associated with PSMA5 promoter, one with WDR47 promoter (pair i-ii), and the other with TAF13 promoter (pair i-iii). The red and blue lines in 3C signals represent two biological replicates. Each biological replicate is averaged from three technical replicates. (**b**) Validation of four distal interactions associated with a lncRNA promoter, three pairs of interactions with predicted enhancers (pair i-ii, i-iv, and i-v, respective), and the fourth with ITGB1 promoter (pair i-iii). (**c**) Validation of interactions between SNX18 promoter and three predicted enhancers (pair i-ii, i-iii, i-iv, respectively). (**d**) Validation of a distal interaction between KATNBL1 and PGBD4 promoters (pair i-ii). (**e**) Validation of a distal interaction between BHLHE40 promoter and a predicted enhancer (pair i-ii).

Supplementary Figure 8

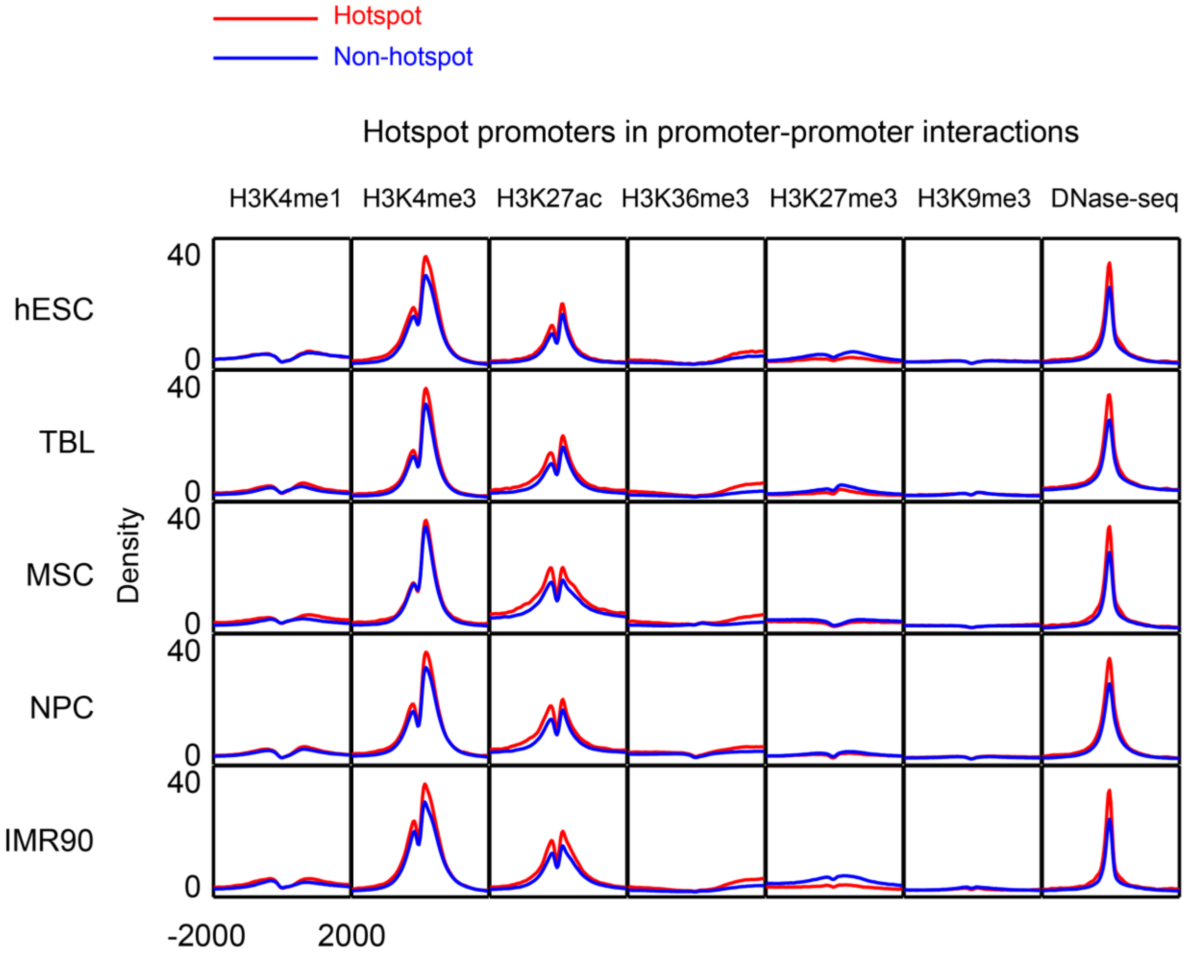

Supplementary Figure 8. Comparison of hotspot and non-hotspot promoters in promoter-promoter interactions in terms of H3K4me1, H3K4me3, H3K27ac, H3K36me3, H3K27me3, H3K9me3, and DNaseI-seq profiles. All promoter profiles were centered on TSSs..

Supplementary Figure 9

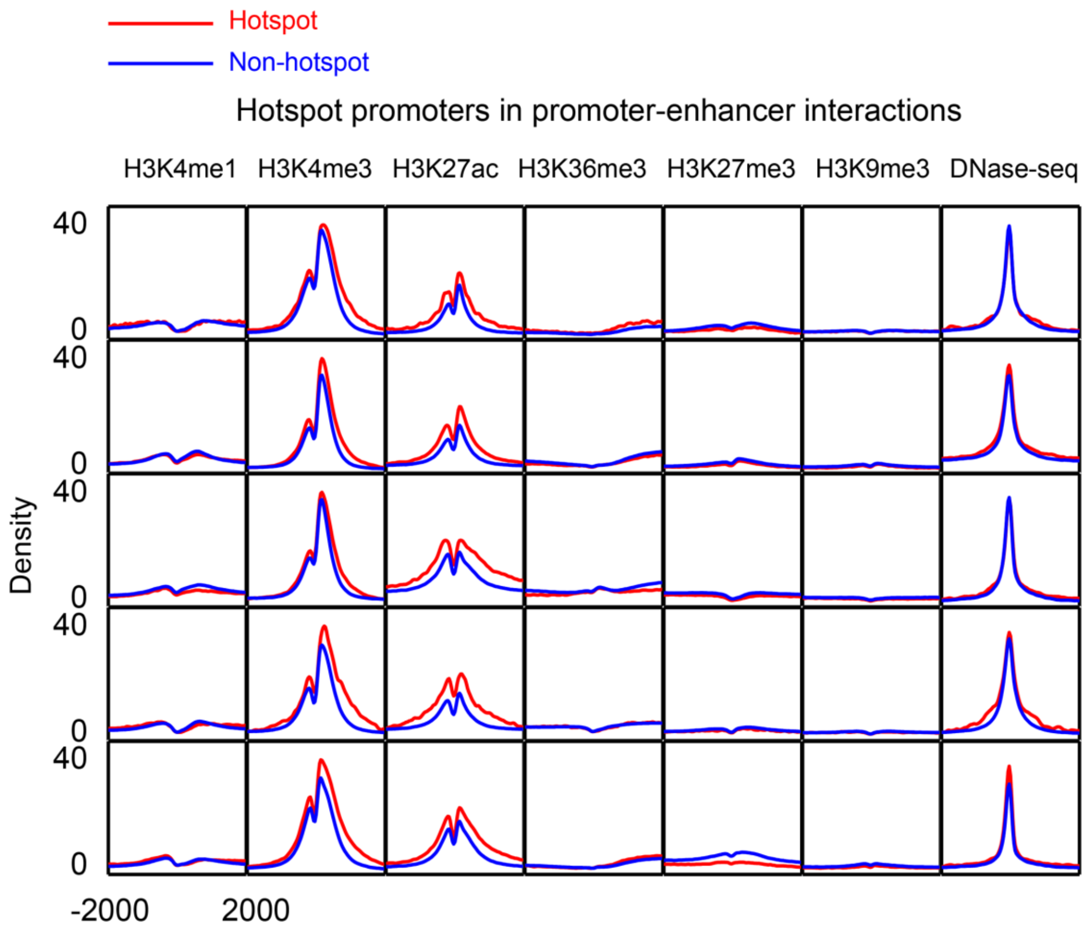

Supplementary Figure 9. Comparison of hotspot and non-hotspot promoters in promoter-enhancer interactions in terms of H3K4me1, H3K4me3, H3K27ac, H3K36me3, H3K27me3, H3K9me3, and DNaseI-seq profiles. All promoter profiles were centered on TSSs.

Supplementary Figure 10

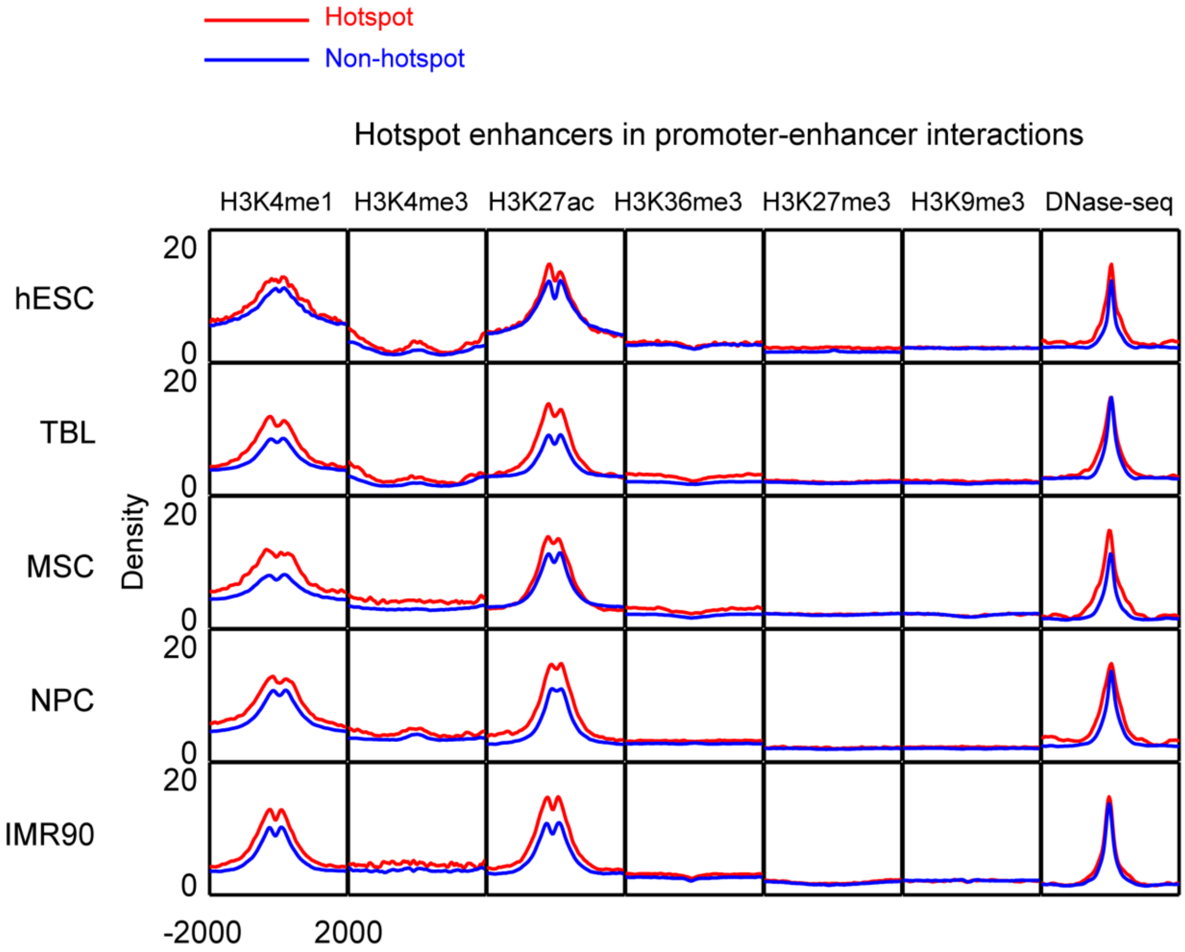

Supplementary Figure 10. Comparison of hotspot and non-hotspot enhancers in promoter-enhancer interactions in terms of H3K4me1, H3K4me3, H3K27ac, H3K36me3, H3K27me3, H3K9me3, and DNaseI-seq profiles. All enhancer profiles were centered on the center of candidate enhancers.

Supplementary Figure 11

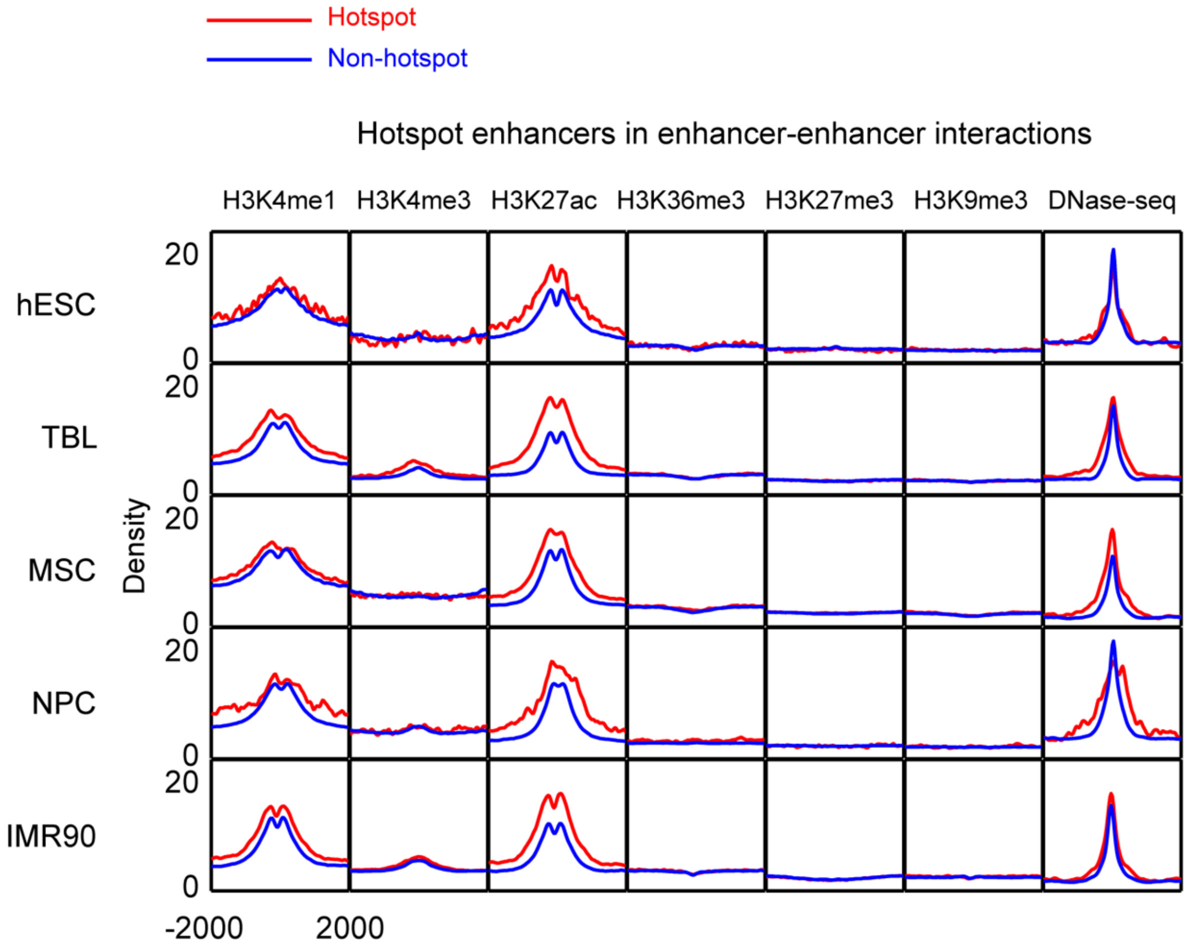

Supplementary Figure 11. Comparison of hotspot and non-hotspot enhancers in enhancer-enhancer interactions in terms of H3K4me1, H3K4me3, H3K27ac, H3K36me3, H3K27me3, H3K9me3, and DNaseI-seq profiles. All enhancer profiles were centered on the center of candidate enhancers.

Supplementary Figure 12

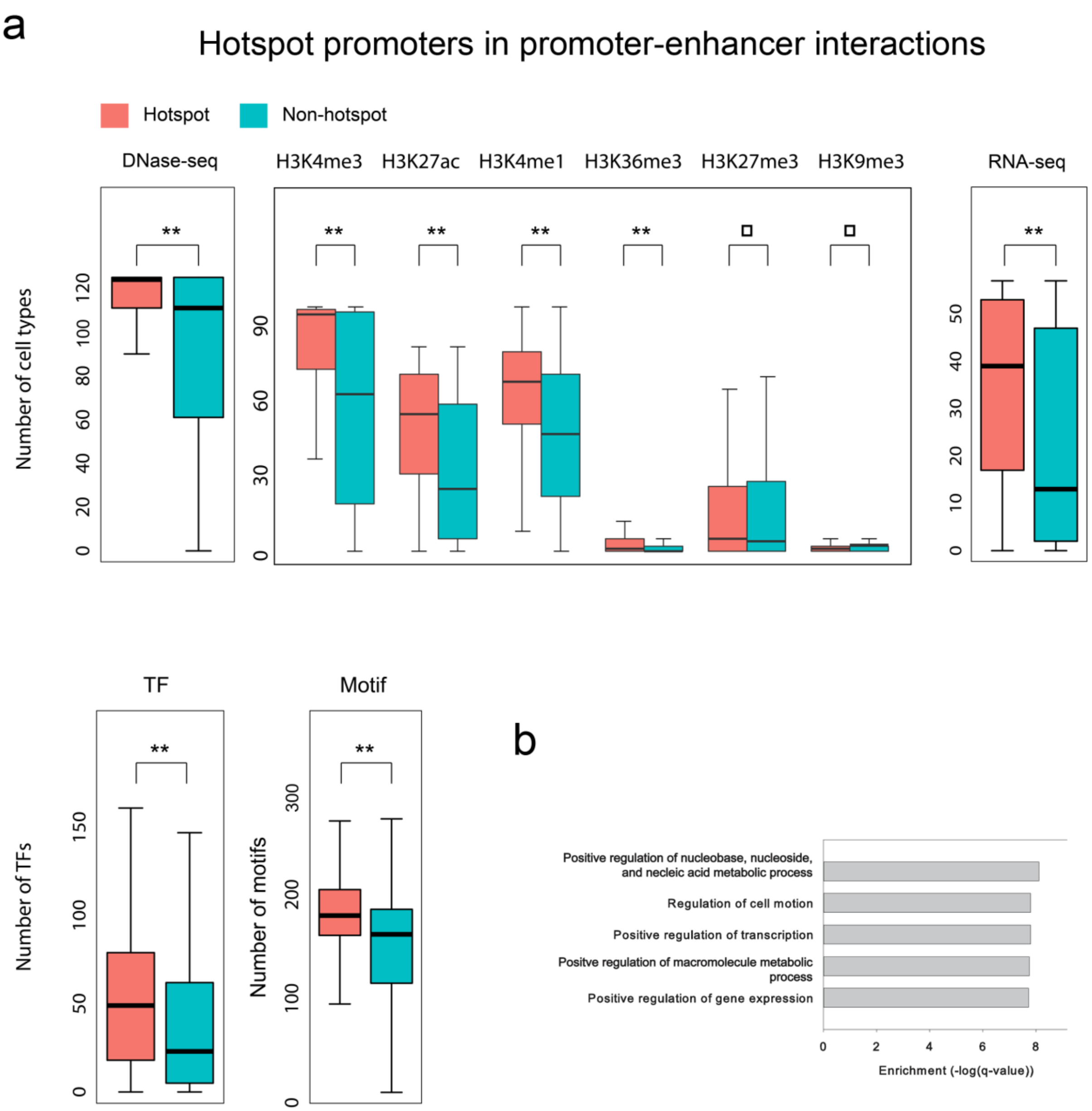

Supplementary Figure 12. Characterization of interaction hotspot promoters in promoter-enhancer interactions. (**a**) Comparison of hotspots and non-hotspots in terms of chromatin accessibility, histone modifications, gene expression, TF binding, and motif enrichment. P-values calculated using Wilcox test are denoted: $** P \leq 0.001$; $*\ 0.001 < P \leq 0.05$; □ $P > 0.05$. (**b)** GO term analysis of hotspot promoters.

Supplementary Figure 13

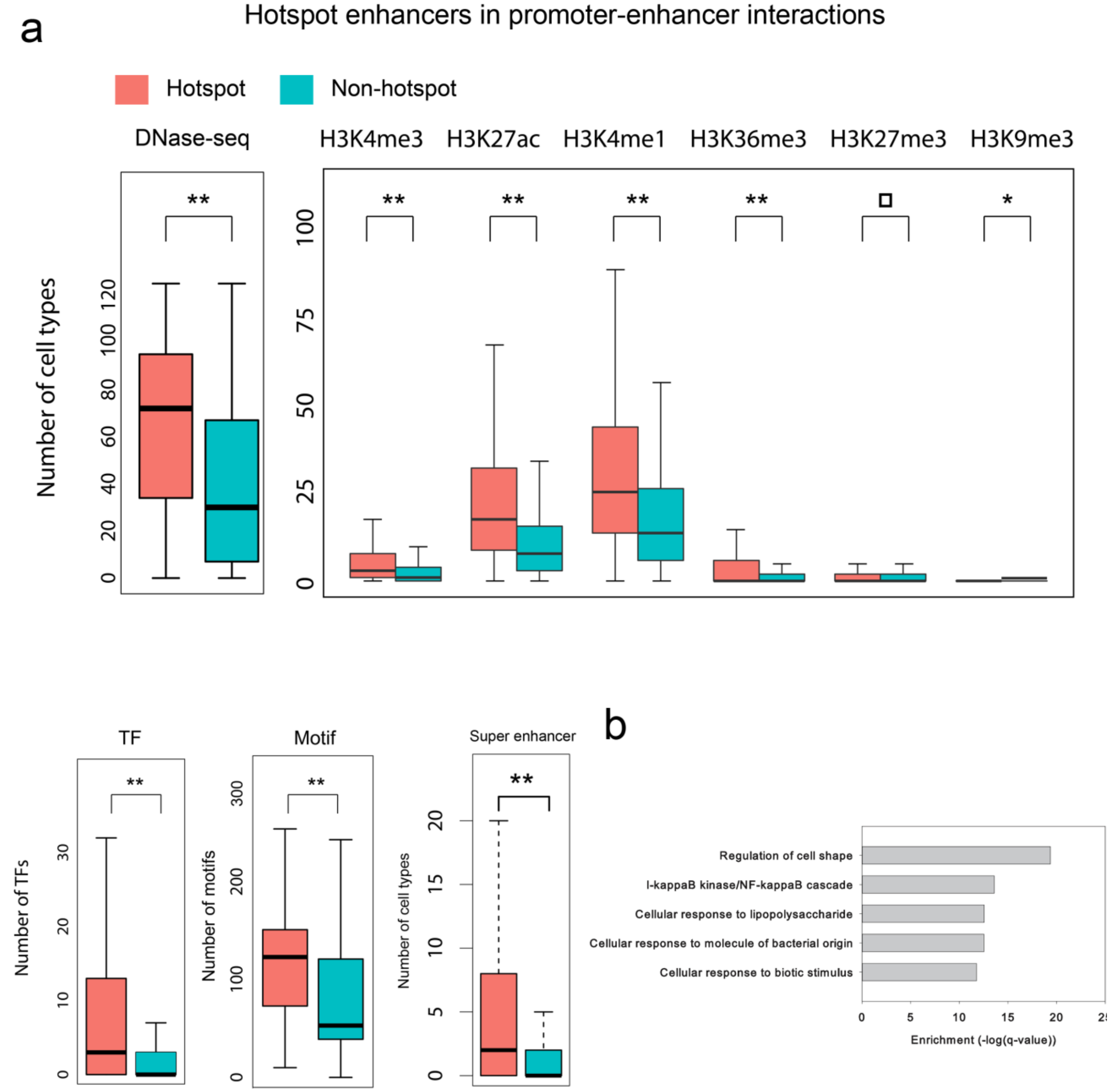

Supplementary Figure 13. Characterization of interaction hotspot enhancers in promoter-enhancer interactions. (**a**) Comparison of hotspots and non-hotspots in terms of chromatin accessibility, histone modifications, TF binding, and motif enrichment. P-values calculated using Wilcox test are denoted: ** $P \le 0.001$; * $0.001 < P \le 0.05$; □ $P > 0.05$. (**b**) GREAT analysis of hotspot enhancers.

Supplementary Figure 14

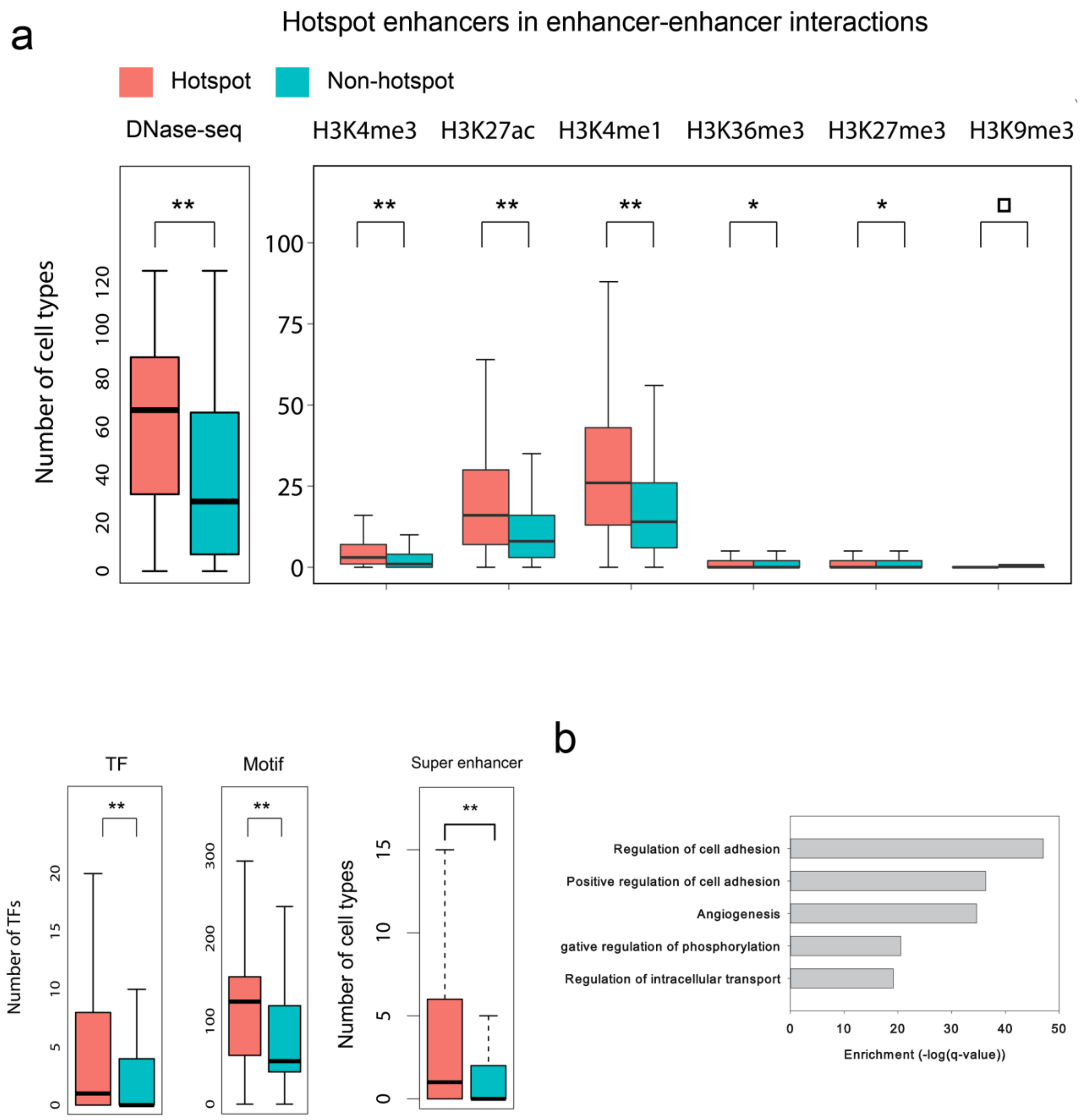

Supplementary Figure 14. Characterization of interaction hotspot enhancers in enhancer-enhancer interactions. (**a**) Comparison of hotspots and non-hotspots in terms of chromatin accessibility, histone modifications, TF binding, and motif enrichment. P-values calculated using Wilcox test are denoted: ** $P \leq 0.001$; * $0.001 < P \leq 0.05$; □ $P > 0.05$. (**b**) GREAT analysis of hotspot enhancers.

Supplementary Table 1 List of six core chromatin modifications and RNA-seq data in 121 cell types

	H3K4me1	H3K4me3	H3K27ac	H3K27me3	H3K36me3	H3K9me3	RNA-seq
ES-I3 Cell Line	X	X		X	X	X	
ES-WA7 Cell Line	X	X		X		X	
H1 Cell Line	X	X	X	X	X	X	X
H1 BMP4 Derived Mesendoderm Cultured Cells	X	X	X	X	X	X	X
H1 BMP4 Derived Trophoblast Cultured Cells	X		X	X	X	X	X
H1 Derived Mesenchymal Stem Cells			X		X	X	X
H1 Derived Neuronal Progenitor Cultured Cells			X		X	X	X
H9 Cell Line			X		X	X	
H9 Derived Neuronal Progenitor Cultured Cells		X			X	X	
H9 Derived Neuron Cultured Cells	X	X		X	X	X	
hESC Derived CD184+ Endoderm Cultured Cells	X	X	X	X	X	X	X
hESC Derived CD56+ Ectoderm Cultured Cells	X	X	X	X	X	X	X
hESC Derived CD56+ Mesoderm Cultured Cells	X	X	X	X	X	X	X
HUES48 Cell Line			X	X	X	X	
HUES6 Cell Line			X		X	X	
HUES64 Cell Line			X		X	X	X
IMR90 fetal lung fibroblasts Cell Line			X		X	X	
iPS-15b Cell Line						X	
iPS-18 Cell Line			X		X	X	
iPS-20b Cell Line			X		X	X	
iPS DF 6.9 Cell Line			X		X	X	
iPS DF 19.11 Cell Line			X		X	X	
Mesenchymal Stem Cell Derived Adipocyte Cultured Cells	X	X			X	X	
ES-UCSF4 Cell Line	X	X		X	X	X	X
Adipose Derived Mesenchymal Stem Cell Cultured Cells	X	X		X	X	X	
Bone Marrow Derived Cultured Mesenchymal Stem Cells	X	X	X	X	X	X	
Breast Myoepithelial Primary Cells	X	X		X	X	X	X
Breast variant Human Mammary Epithelial Cells (vHMEC)	X	X		X	X	X	X
Primary monocytes from peripheral blood	X	X	X	X	X	X	
Primary neutrophils from peripheral blood	X	X		X	X	X	
Primary B cells from cord blood	X	X		X	X	X	
Primary B cells from peripheral blood	X	X	X	X	X	X	
Primary T cells from cord blood	X	X		X	X	X	
Primary T cells from peripheral blood	X	X	X	X		X	
Primary hematopoietic stem cells	X	X		X	X	X	
Primary hematopoietic stem cells short term culture	X	X		X		X	

Primary T helper memory cells from peripheral blood 2	X	X	X	X		X	X
Primary T helper naive cells from peripheral blood	X	X	X	X	X	X	X
Primary T helper naive cells from peripheral blood	X	X	X	X	X	X	
Primary T helper memory cells from peripheral blood 1	X	X	X	X	X	X	
Primary T helper cells PMA-I stimulated	X	X	X	X	X	X	
Primary T helper 17 cells PMA-I stimulated	X	X	X	X		X	
Primary T helper cells from peripheral blood	X	X	X	X	X	X	
Primary T regulatory cells from peripheral blood	X	X	X	X	X	X	
Primary T cells effector/memory enriched from peripheral blood	X	X	X	X	X	X	
Primary Natural Killer cells from peripheral blood	X	X	X	X		X	
Primary T CD8+ naïve cells from peripheral blood	X	X	X	X	X	X	X
Primary T CD8+ memory cells from peripheral blood	X	X	X	X	X	X	
Mesenchymal Stem Cell Derived Chondrocyte Cultured Cells	X	X	X	X	X	X	
Primary hematopoietic stem cells G-CSF-mobilized Female	X	X	X	X	X	X	X
Primary hematopoietic stem cells G-CSF-mobilized Male	X	X		X	X	X	
Muscle Satellite Cultured Cells	X	X		X	X	X	
Cortex derived primary cultured neurospheres	X	X		X	X	X	X
Ganglion Eminence derived primary cultured neurospheres	X	X		X	X	X	X
Foreskin Fibroblast Primary Cells skin01	X	X	X	X		X	X
Foreskin Fibroblast Primary Cells skin02	X	X	X	X	X	X	X
Foreskin Keratinocyte Primary Cells skin02	X	X		X	X	X	X
Foreskin Keratinocyte Primary Cells skin03	X	X	X	X	X	X	X
Foreskin Melanocyte Primary Cells skin01	X	X	X	X	X	X	X
Foreskin Melanocyte Primary Cells skin03	X	X	X	X	X	X	X
Primary mononuclear cells from peripheral blood	X	X	X	X	X	X	X
Adipose Nuclei	X	X	X	X		X	
Aorta	X	X	X	X	X	X	X
Liver	X	X	X	X	X	X	X
Brain Angular Gyrus	X	X	X	X	X	X	
Brain Anterior Caudate	X	X	X	X	X	X	
Brain Cingulate Gyrus	X	X	X	X		X	
Brain Germinal Matrix	X	X		X	X	X	X
Brain Hippocampus Middle	X	X	X	X	X	X	X
Brain Inferior Temporal Lobe	X	X	X	X	X	X	
Brain Dorsolateral Prefrontal Cortex	X	X	X	X		X	
Brain Substantia Nigra	X	X	X	X	X	X	
Colonic Mucosa	X	X	X	X	X	X	
Colon Smooth Muscle	X	X	X	X		X	
Duodenum Mucosa	X	X		X	X	X	
Duodenum Smooth Muscle	X	X	X	X	X	X	
Esophagus	X	X	X	X	X	X	X

Fetal Adrenal Gland	X	X	X	X	X	X	
Fetal Brain Male	X	X		X	X	X	
Fetal Brain Female	X	X		X	X	X	X
Fetal Heart	X	X		X	X	X	
Fetal Intestine Large	X	X	X	X	X	X	X
Fetal Intestine Small	X	X	X	X	X	X	X
Fetal Kidney	X	X		X	X	X	
Pancreatic Islets	X	X	X	X	X	X	X
Fetal Lung	X	X		X	X	X	
Fetal Muscle Trunk	X	X	X	X		X	
Fetal Muscle Leg	X	X	X	X	X	X	
Placenta	X	X	X	X	X	X	
Fetal Stomach	X	X	X	X		X	
Fetal Thymus	X	X	X	X	X	X	
Gastric	X	X	X	X	X	X	X
Left Ventricle	X	X	X	X	X	X	X
Lung	X	X	X	X	X	X	X
Ovary	X	X	X	X	X	X	X
Pancreas	X	X	X	X	X	X	X
Placenta Amnion	X	X	X	X		X	
Psoas Muscle	X	X	X	X		X	X
Rectal Mucosa Donor 29	X	X	X	X	X	X	
Rectal Mucosa Donor 31	X	X	X	X	X	X	
Rectal Smooth Muscle	X	X	X	X	X	X	
Right Atrium	X	X	X	X		X	X
Right Ventricle	X	X	X	X	X	X	X
Sigmoid Colon	X	X	X	X	X	X	X
Skeletal Muscle Male	X	X		X		X	
Skeletal Muscle Female	X	X	X	X	X	X	
Small Intestine	X	X	X	X	X	X	X
Stomach Mucosa	X	X		X		X	
Stomach Smooth Muscle	X	X	X	X	X	X	
Thymus	X	X	X	X		X	X
Spleen	X	X	X	X		X	X
A549 EtOH 0.02pct Lung Carcinoma Cell Line							X
GM12878 Lymphoblastoid Cell Line							X
HeLa-S3 Cervical Carcinoma Cell Line							X
HepG2 Hepatocellular Carcinoma Cell Line							X
HMEC Mammary Epithelial Primary Cells							X
HSMM Skeletal Muscle Myoblasts Cell Line							X
HUVEC Umbilical Vein Endothelial Cells Cell Line							X

K562 Leukemia Cell Line							X
NHEK-Epidermal Keratinocyte Primary Cells							X
NHLF Lung Fibroblast Primary Cells							X

Suppplementary Table 2 List of DNase-seq data in 125 cell types

1	8988T	52	CMK	103	RPTEC
2	AoSMC	53	GM06990	104	SAEC
3	Chorion	54	GM12864	105	SKMC
4	CLL	55	GM12865	106	SK-N-MC
5	Fibrobl	56	H7-hESC	107	SK-N-SH_RA
6	FibroP	57	HAc	108	Th2
7	Gliobla	58	HAEpiC	109	WERI-Rb-1
8	GM12891	59	HA-h	110	WI-38
9	GM12892	60	HA-sp	111	WI-38
10	GM18507	61	HBMEC	112	A549
11	GM19238	62	HCF	113	GM12878
12	GM19239	63	HCFaa	114	H1-hESC
13	GM19240	64	HCM	115	HeLa-S3
14	H9ES	65	HConF	116	HepG2
15	HeLa-S3	66	HCPEpiC	117	HMEC
16	Hepatocytes	67	HCT-116	118	HSMM
17	HPDE6-E6E7	68	HEEpiC	119	HSMMtube
18	HSMM_emb	69	HFF	120	HUVEC
19	HTR8svn	70	HFF-Myc	121	K562
20	Huh-7.5	71	HGF	122	LNCaP
21	Huh-7	72	HIPEpiC	123	MCF-7
22	iPS	73	HL-60	124	NHEK
23	Ishikawa	74	HMF	125	Th1
24	Ishikawa	75	HMVEC-dAd		
25	LNCaP	76	HMVEC-dBl-Ad		
26	MCF-7	77	HMVEC-dBl-Neo		
27	Medullo	78	HMVEC-dLy-Ad		
28	Melano	79	HMVEC-dLy-Neo		
29	Myometr	80	HMVEC-dNeo		
30	Osteobl	81	HMVEC-LBl		
31	PanIsletD	82	HMVEC-LLy		
32	PanIslets	83	HNPCEpiC		
33	pHTE	84	HPAEC		
34	ProgFib	85	HPAF		
35	RWPE1	86	HPdLF		
36	Stellate	87	HPF		
37	T-47D	88	HRCEpiC		
38	Adult_CD4_Th0	89	HRE		
39	Urothelia	90	HRGEC		
40	Urothelia	91	HRPEpiC		

41	AG04449	92	HVMF		
42	AG04450	93	Jurkat		
43	AG09309	94	Monocytes-CD14+_RO01746		
44	AG09319	95	NB4		
45	AG10803	96	NH-A		
46	AoAF	97	NHDF-Ad		
47	BE2_C	98	NHDF-neo		
48	BJ	99	NHLF		
49	Caco-2	100	NT2-D1		
50	CD20+	101	PANC-1		
51	CD34+_Mobilized	102	PrEC		

Supplementary Table 3 List of TF ChIP-seq data in ENCODE Tier1 cells	
	TFs
hESC (49)	ARID3A,ATF2,ATF3,BACH1,BCL11A,BRCA1,CEBPB,CHD1,CHD2,CTBP2,CTCF,EGR1,EP300,EZH2,FOS,GABPA,GTF2B,HDAC2,JUN,JUND,MAFK,MAX,MXI1,MYC,NANOG,NRF1,NRSF,POL2,POU5F1,RAD21,RBBP5,RFX5,RXRA,SIN3A,SIX5,SP1,SP2,SP4,SRF,SUZ12,TAF1,TAF7,TBP,TCF12,TEAD4,USF1,USF2,YY1,ZNF143
GM12878 (77)	ATF2,ATF3,BATF,BCL11A,BCL3,BCLAF1,BHLHE40,BRCA1,CEBPB,CHD1,CHD2,CORES,CTCF,E2F4,EBF1,EGR1,ELF1,ELK1,ETS1,EZH2,FOS,FOXM1,GABPA,IKZF1,IRF4,JUND,MAX,MAZ,MEF2A,MEF2C,MTA3,MXI1,MYC,NFATC1,NFE2,NFIC,NFYA,NFYB,NR2C2,NRF1,P300,PAX5,PBX3,PML,POL2,POLR2A,POLR3G,POU2F2,RAD21,RELA,REST,RFX5,RUNX3,RXRA,SIN3A,SIX5,SMC3,SP1,SPI1,SRF,STAT1,STAT3,STAT5A,TAF1,TBL1XR1,TBP,TCF12,TCF3,USF1,USF2,WRNIP1,YY1,ZBTB33,ZEB1,ZNF143,ZNF274,ZZZ3
K562(98)	ARID3A,ATF1,ATF3,BACH1,BCL3,BCLAF1,BDP1,BHLHE40,BRF1,BRF2,CBX3,CCNT2,CEBPB,CHD1A,CHD2,CTCF,E2F4,E2F6,EGR1,ELF1,ELK1,ETS1,EZH2,FOS,FOSL1,GABPA,GATA1,GATA2,GTF2B,GTF2F1,GTF3C2,HDAC1,HDAC2,HDAC6,HDAC8,HMGN3,IRF1,JUN,JUND,KAP1,KDM5B,MAFF,MAFK,MAX,MAZ,MEF2A,MXI1,MYC,NFE2,NFYA,NFYB,NR2C2,NR2F2,NRF1,P300,PHF8,PML,POL2,POL2,POL3,RAD21,RBBP5,RCOR1,REST,RFX5,RPC155,SAP30,SETDB1,SIN3A,SIRT6,SIX5,SMARCA4,SMARCB1,SMC3,SP1,SP2,SPL1,SRF,STAT1,STAT2,STAT5A,TAF1,TAF7,TAL1,TBL1XR1,TBP,TEAD4,THAP1,TRIM28,UBTF,USF1,USF2,YY1,ZBTB33,ZBTB7A,ZNF143,ZNF263,ZNF274

Supplementary Table 4 Primer sequences for 3C experiments

Strand	Primer	Chromosome coordinates				Sequence (5'->3')	Length
-	HiCN1-H-Anchor	chr1:	109965720	-	109965740	CTGGACTCCATAAACCACGGA	21
-	HiCN1-H-1	chr1:	109567581	-	109567600	TCTGTTGTTCAGAGGTGTGC	20
-	HiCN1-H-2	chr1:	109570093	-	109570115	TGGGAGCCTGTATAAATGTTTCA	23
-	HiCN1-H-3	chr1:	109581012	-	109581031	ACTGCACCTGGCTCGATATT	20
-	HiCN1-H-4	chr1:	109582408	-	109582426	CTTGGGCTCGCAATGTTGG	19
-	HiCN1-H-5	chr1:	109582930	-	109582949	GGGCGGCATATGAAATGCTT	20
-	HiCN1-H-6	chr1:	109593464	-	109593483	AGTGGTGGTGCTTTGTTCTC	20
-	HiCN1-H-7	chr1:	109600737	-	109600757	TAGGCAACACACCACTATGCC	21
-	HiCN1-H-8	chr1:	109605594	-	109605615	GAATAATGCTGTGTTGGAAGGC	22
+	HiCN1-H-9	chr1:	109625003	-	109625022	GAAAGCAGGAGCTGGATGGT	20
-	HiCN1-H-10	chr1:	109625134	-	109625153	AGACGTCTACACCCTGGGAT	20
+	HiCN1-IC-F	chr1:	109969222	-	109969241	CTTAAATCGCGGGTGGTTGC	20
-	HiCN1-IC-R	chr1:	109969459	-	109969478	ACCGTGGAGGAACCCTAAGA	20
+	HiCP1-H-Anchor	chr1:	32400038	-	32400058	GAGAGGAGCAGGTGCATACAT	21
+	HiCP1-H-1	chr1:	32464186	-	32464206	TGCAGACCAACCATGCTGATA	21
+	HiCP1-H-2	chr1:	32468909	-	32468929	TTCTACAGTGAGGTCCACAGC	21
+	HiCP1-H-3	chr1:	32473194	-	32473214	GGGATGCAATTGGCTTTCTGG	21
+	HiCP1-H-4	chr1:	32477638	-	32477662	TACGTGTTTTGTTTTCATTTCTGTG	25
+	HiCP1-H-5	chr1:	32487016	-	32487038	AGAACCTATTGGCTTTGTGCTCT	23
+	HiCP1-H-6	chr1:	32488684	-	32488704	ACACCAAGCATGTAGGAGAGC	21
+	HiCP1-H-7	chr1:	32496011	-	32496031	GCAGTATCCCAAGGTAAGGCA	21
+	HiCP1-H-8	chr1:	32499290	-	32499312	GGAAAGTGACTAAGGTGGGTCTC	23
+	HiCP1-IC-F	chr1:	32402503	-	32402522	CGACTCCGGACAGACAAACA	20
-	HiCP1-IC-R	chr1:	32402730	-	32402749	GCCCCTGAGAGCTAACGAAA	20
-	HiCP10-H-Anchor	chr10:	33293318	-	33293337	TCAGGAAGAGGGAAAGGACT	20
-	HiCP10-H-1	chr10:	33225735	-	33225757	TCTTCTTGTGGAGGGACAGATAC	23
-	HiCP10-H-2	chr10:	33233791	-	33233814	GTCTCTGGTTTATCATCCTCATGC	24
-	HiCP10-H-3	chr10:	33233958	-	33233979	CAAGGAATAGGAGGTTTGCTCA	22
-	HiCP10-H-4	chr10:	33237577	-	33237599	CTCCGTCTTTTATCTGGAACCCA	23
-	HiCP10-H-5	chr10:	33241680	-	33241704	GGAGATGAAGAGGCAATAAAGACAG	25

-	HiCP10-H-6	chr10:	33243412	-	33243436	TTGCATTTATTTTGGGTACAGGTAT	25
-	HiCP10-H-7	chr10:	33246256	-	33246275	CGCGTGTCATTTCTTAGGCT	20
-	HiCP10-H-8	chr10:	33248579	-	33248601	ACAGTTTTCTGTGCTGAGACTGG	23
-	HiCP10-H-9	chr10:	33254910	-	33254929	ATGTTTAGGGTGTCCTACGC	20
-	HiCP10-H-10	chr10:	33255816	-	33255835	CCTGGGTGACAAAAGCGAGA	20
-	HiCP10-H-11	chr10:	33259729	-	33259751	GGAGGCTAGATCCAAGTTAGTGG	23
-	HiCP10-H-12	chr10:	33260230	-	33260251	CAGAAAGAGATTCCAGGCTTGC	22
-	HiCP10-H-13	chr10:	33266204	-	33266227	ACAGCTTTTAGATCACACTTTGAA	24
-	HiCP10-H-14	chr10:	33272604	-	33272623	GGCTGCATGGTGGCTTTATC	20
-	HiCP10-H-15	chr10:	33274915	-	33274936	TGGAAAACAGGCATTCAGCCTA	22
+	HiCP10-IC-F	chr10:	33294482	-	33294501	TAATGCTCCCAAGCAGAGGC	20
-	HiCP10-IC-R	chr10:	33294702	-	33294721	CAGTGCAAACGCAGTGAGTC	20
+	HiCN11-H-Anchor	chr11:	82614994	-	82615014	TTTGGGCACAGTCTTGAAGCA	21
+	HiCN11-H-1	chr11:	82762882	-	82762901	AGCTCTGCTGTCCCAGAATC	20
+	HiCN11-H-2	chr11:	82768391	-	82768412	ACCCAACTTCCAATTCCTGTAG	22
+	HiCN11-H-3	chr11:	82768965	-	82768984	CCATCCTACAATGCCAGCCT	20
+	HiCN11-H-4	chr11:	82773006	-	82773026	TGGCTCCAGTGAACAAAAGGT	21
+	HiCN11-H-5	chr11:	82783558	-	82783577	CCCACAGAGTTGGGGTAGTC	20
+	HiCN11-H-6	chr11:	82784802	-	82784824	ACAGGAAAAGGAGCAATGTCTAC	23
+	HiCN11-H-7	chr11:	82785058	-	82785081	GCAACAGATTACTCAGAAACCTTC	24
+	HiCN11-H-8	chr11:	82785412	-	82785431	ATTTGAGGAGTTGGGGAGGC	20
+	HiCN11-H-9	chr11:	82786051	-	82786071	ACAAGAGTTGGGTGGAGTAGT	21
+	HiCN11-H-10	chr11:	82787288	-	82787309	GCTTCAGATACTTCAGAGTGGT	22
+	HiCN11-H-11	chr11:	82793219	-	82793238	TTCCCTGGAGGGACAGAACT	20
+	HiCN11-H-12	chr11:	82795265	-	82795284	AGGTTGCAGTACAGTGAGCC	20
+	HiCN11-H-13	chr11:	82796463	-	82796482	GGATGAGGTTGGGGGAGGAT	20
+	HiCN11-IC-F	chr11:	82612898	-	82612917	ACTCTACCCGCAGTTTGACG	20
-	HiCN11-IC-R	chr11	82612628	-	82612647	CCTTAGGAGTCTCCGGCTCT	20

		:					
-	HiCN15-H-Anchor	chr15:	34500979	-	34500999	AAACTTCGGAGACCTGAGTGT	21
-	HiCN15-H-1	chr15:	34385921	-	34385944	TCTGTGCCTTAAAGGACTTATTCA	24
-	HiCN15-H-2	chr15:	34389695	-	34389719	GCTCTTGATAGGATGGTTTTCTAAG	25
-	HiCN15-H-3	chr15:	34392741	-	34392761	TCTGGCATTAGGGCATTCGTT	21
-	HiCN15-H-4	chr15:	34399518	-	34399541	AGAATCAGTCCATCCATAAGCTGG	24
-	HiCN15-H-5	chr15:	34406550	-	34406571	ACTCTACCCACAGGACATGAGT	22
-	HiCN15-H-6	chr15:	34413561	-	34413580	GGGTGACAGAGTGAGACCCT	20
+	HiCN15-IC-F	chr15:	34502132	-	34502150	CCGGCTCGGCAGTCACTTA	19
-	HiCN15-IC-R	chr15:	34502408	-	34502427	AAGGCTACCGTGCGAAGCTC	20
-	PE3-H-Anchor	chr3:	5018711	-	5018730	GAGAGGACAGGACACGTACC	20
-	PE3-H-1	chr3:	4744920	-	4744939	TCCCAGGGCCTTGAATAAGC	20
-	PE3-H-2	chr3:	4745326	-	4745346	TGCTGAGAACTACACCTGTCT	21
-	PE3-H-3	chr3:	4752282	-	4752302	GGGAGGTCAGAACAGGACTTT	21
-	PE3-H-4	chr3:	4753140	-	4753159	CCACTCAGACCTGCTCGTTT	20
-	PE3-H-5	chr3:	4756057	-	4756077	GGAAGTGTCAGTCATGGGACA	21
-	PE3-H-6	chr3:	4761209	-	4761229	TACGCAGTTCAGATGTGAGGC	21
-	PE3-H-7	chr3:	4763285	-	4763304	AGCCGCTCGAATCAGTCTTC	20
-	PE3-H-8	chr3:	4768169	-	4768193	AGCTTGAACTACTGTGATTTTGGAA	25
-	PE3-H-9	chr3:	4771767	-	4771790	TGTGAAAACTTGGGTTCAAGGAAA	24
-	PE3-H-10	chr3:	4775448	-	4775470	ATGTACACAGCATGGGCATTAGA	23
-	PE3-H-11	chr3:	4778018	-	4778038	ACGCCCTTTACAAAGTTGCAC	21
+	PE3-IC-F	chr3:	5024799	-	5024818	CCAGTCATCCAGCGGACTTT	20
-	PE3-IC-R	chr3:	5024976	-	5024995	GGGGGTTCTTCGGACTCTTG	20
+	PE5-H-Anchor	chr5:	53817956	-	53817976	CAGTGTGCGATACATAAGGCT	21
+	PE5-H-1	chr5:	54120366	-	54120387	TCACCAGCCAATTCCTTACTGG	22
+	PE5-H-2	chr5:	54121601	-	54121620	TTTGGGCACCAAGGTGACTG	20
+	PE5-H-3	chr5:	54123573	-	54123592	CCCATCAGCCAATTTCCAGC	20
+	PE5-H-4	chr5:	54128070	-	54128090	AGCCACATTCAACTTTGCCAC	21
+	PE5-H-5	chr5:	54130037	-	54130056	TTAGGCCTTCGCAGGATAGC	20
+	PE5-H-6	chr5:	54136550	-	54136569	GCACTGCTAGGTGCTAAGGA	20
+	PE5-H-7	chr5:	54146368	-	54146387	GGCAGCTCTACTGCTTCTCC	20
+	PE5-H-8	chr5:	54152407	-	54152427	TGGCTGTTACCAATGGAGCTT	21
+	PE5-H-9	chr5:	54153203	-	54153225	GGATGGGAACTGTGTGACTGTAA	23
+	PE5-H-10	chr5:	54160192	-	54160211	ACAGGGCCAGTGACAATGTG	20
+	PE5-H-11	chr5:	54166834	-	54166853	CTTGATGACCTCAGGGACGG	20
+	PE5-H-12	chr5:	54168865	-	54168885	ACTGTCACTGGTGGTAGGTGA	21
+	PE5-H-13	chr5:	54172664	-	54172683	CAAATGCACACAGGTCGATG	20
+	PE5-IC-F	chr5:	53812629	-	53812648	TGGTGCATGTACCCTGCAAT	20
-	PE5-IC-R	chr5:	53812960	-	53812979	CATGTGTCGCCTATCCTCCC	20

Supplemental Note 1 - Tensor Basics

1. Introduction

Often the input for machine learning problems is inherently multi-dimensional. For example, gray-scale video sequences are three-dimensional: column, row, and time [1, 2]. Color video sequences are four-dimensional, as there is an additional color dimension [3, 4]. Data in social networks are usually organized in three dimensions: time, authors, and keywords [5]. These multidimensional data can be formally represented as tensors, the elements of which are addressed by more than two indices. The number of indices used in the description defines the order of the tensor and each index defines one of the so-called "mode". Tensors can be viewed as an extension of matrices, vectors, and scalars. A matrix is a second-order tensor, a vector a first-order tensor, and a scalar zeroth-order tensor. Following the notations used in [6, 7], we denote tensors by calligraphic letters, matrices by uppercase boldface letters, and vectors by lowercase-boldface letters.

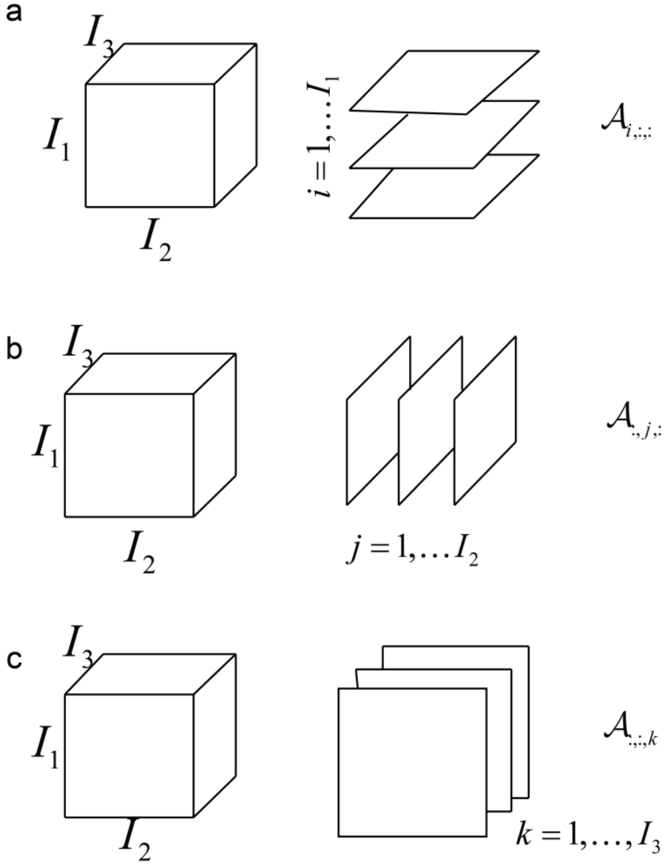

Figure 1. Slices of a 3rd-order tensor.

Mathematically, an Nth-order tensor is denoted as $\mathcal{A}$ with mode $I_1 \times I_2 \times \ldots \times I_N$. The n-th mode of $\mathcal{A}$ is of size I_n. We denote the index of a single element within a tensor by subscripts (e.g. $\mathcal{A}_{i_1 i_2 \ldots i_N}$). We use colon notation to denote the full range of a given index. Consider a 3rd-

order tensor, specifying a single index yields a slice. $A_{i,:,:}$ yields the i th horizontal slice, $A_{:,j,:}$ the j th lateral slice, and $A_{:,:,k}$ the k th frontal slice (Fig. 1). Similarly, specifying two indices in a 3rd –order tensor yields a vector. $A_{:,j,k}$ yields a column vector, $A_{i,:,k}$ yields a row vector, and $A_{i,j,:}$ yields a tube vector.

2. Matricization of a tensor

Matricization of a tensor refers to the process of rearranging the elements of a tensor to form a matrix. It is also known as an “unfolding” operation and is defined to a specific mode. For order three, the matricization process is visualized in Fig. 2. In general, we have the following definition.

Let A be a tensor of mode $I_1 \times I_2 \times \ldots \times I_N$, and suppose we wish to rearrange it to be a matrix $\mathbf{A}_{(n)}$ with the size of $I_n \times \left(I_{n+1} I_{n+1} \ldots I_N I_1 I_2 \ldots I_{n-1}\right)$. $\mathbf{A}_{(n)}$ contains the element $a_{i_1 i_2 \ldots i_N}$ of A at the position with row number i_n and column number equal to

$$\left(i_{n+1}-1\right) I_{n+2} I_{n+3} \ldots I_N I_1 I_2 \ldots I_{n-1} + \left(i_{n+2}-1\right) I_{n+3} I_{n+4} \ldots I_N I_1 I_2 \ldots I_{n-1} + \ldots$$
$$+ (i_N - 1) I_1 I_2 \ldots I_{n-1} + \left(i_1 - 1\right) I_2 I_3 \ldots I_{n-1} + \left(i_2 - 1\right) I_3 I_4 \ldots I_{n-1} + \ldots i_{n-1}$$

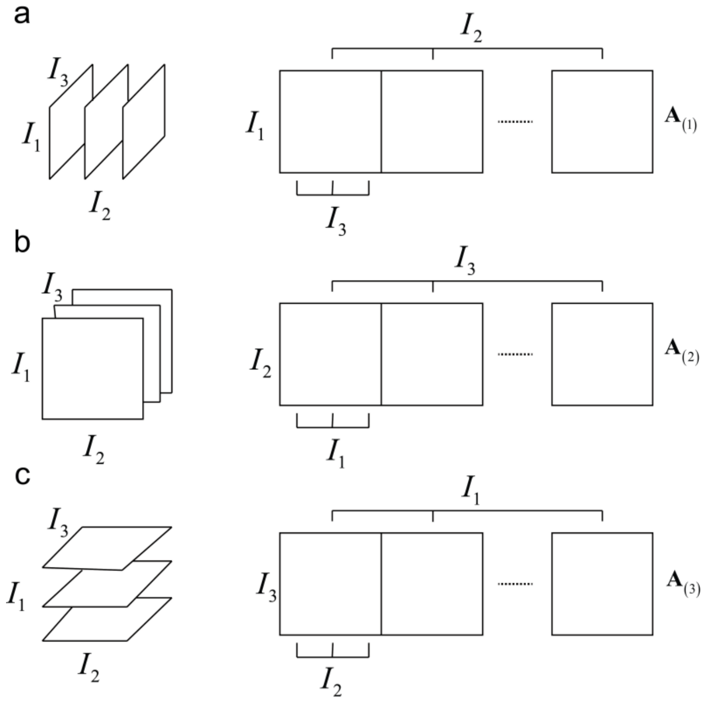

Figure 2. Matricizing a 3rd-order tensor.

3. Norm and inner product of a tensor

Let A and B be two tensors of mode $I_1 \times I_2 \times \ldots \times I_N$, the inner product $\langle A, B \rangle$ is given by

$$\langle A, B \rangle \overset{\text{def}}{=} \sum_{i_1} \sum_{i_2} \cdots \sum_{i_N} A_{i_1 i_2 \ldots i_N} B^{*}_{i_1 i_2 \ldots i_N} ,$$

in which * denotes the complex conjugate.

Using the definition of inner product, the Frobenius-norm of a tensor $\mathcal{A}$ is given by

$$\|\mathcal{A}\|_F \overset{\text{def}}{=} \sqrt{\langle \mathcal{A}, \mathcal{A} \rangle} = \sqrt{\sum_{i_1} \sum_{i_2} \cdots \sum_{i_N} \mathcal{A}^2_{i_1 i_2 \ldots i_N}}$$

4. Tensor multiplication: the *n*-mode product

The *n*-mode product defines multiplication of a tensor by a matrix in mode *n*. Before we introduce the definition of *n*-mode product, let us first take a look at the matrix product $\mathbf{F} = \mathbf{U} \cdot \Sigma \cdot \mathbf{V}^T$, where $\mathbf{F}$ is $J_1 \times J_2$, Σ is $I_1 \times I_2$, $\mathbf{U}$ is $J_1 \times I_1$, and $\mathbf{V}$ is $J_2 \times I_2$. In this matrix multiplication, the columns of Σ is multiplied by $\mathbf{U}$ and the rows of Σ is multiplied by $\mathbf{V}$. Let us denote column multiplication by $\times_1$ and row multiplication by $\times_2$, $\mathbf{F} = \mathbf{U} \cdot \Sigma \cdot \mathbf{V}^T$ can be rewritten as $\mathbf{F} = \Sigma \times_1 \mathbf{U} \times_2 \mathbf{V}$. In general, we have the following definition.

Let $\mathcal{A}$ be a tensor of $I_1 \times I_2 \times \ldots \times I_N$ and $\mathbf{U}$ a matrix of $J_n \times I_n$, the *n*-mode product $\mathcal{A} \times_n \mathbf{U}$ is a $\left(I_1 \times I_2 \times \ldots \times I_{n-1} \times J_n \times I_{n+1} \times \ldots \times I_N\right)$-tensor given by

$$\left(\mathcal{A} \times_n \mathbf{U}\right)_{i_1 i_2 \ldots i_{n-1} j_n i_{n+1} \ldots i_N} \overset{\text{def}}{=} \sum_{i_n} \mathcal{A}_{i_1 i_2 \ldots i_{n-1} i_n i_{n+1} \ldots i_N} \mathbf{U}_{j_n i_n} .$$

The *n*-mode product satisfies the following properties.

a. Let $\mathcal{A}$ be a tensor of $I_1 \times I_2 \times \ldots \times I_N$, $\mathbf{U}$ a matrix of $J_m \times I_m$, and $\mathbf{V}$ a matrix of $J_n \times I_n$,
$\mathcal{A} \times_m \mathbf{U} \times_n \mathbf{V} = \left(\mathcal{A} \times_m \mathbf{U}\right) \times_n \mathbf{V} = \left(\mathcal{A} \times_n \mathbf{V}\right) \times_m \mathbf{U}$

b. Let $\mathcal{A}$ be a tensor of $I_1 \times I_2 \times \ldots \times I_N$, $\mathbf{U}$ a matrix of $J_n \times I_n$, and $\mathbf{V}$ a matrix of $K_n \times J_n$,
$\left(\mathcal{A} \times_n \mathbf{U}\right) \times_n \mathbf{V} = \mathcal{A} \times_n \left(\mathbf{V} \cdot \mathbf{U}\right)$

5. Tensor decomposition

A matrix $\mathbf{A}$ of size $I_1 \times I_2$ is a two-mode mathematical object that has two associated vector spaces, a row space and a column space. Singular value decomposition (SVD) orthogonalizes these two spaces and decomposes the matrix as $\mathbf{A} = \mathbf{U}_1 \Sigma \mathbf{U}_2^T$, the product of an orthogonal column-space represented by the left matrix $\mathbf{U}_1$ of the size $I_1 \times J_1$, a singular value matrix Σ of the size $J_1 \times J_2$, and an orthogonal row space represented by the right matrix $\mathbf{U}_2$ of the size $I_2 \times J_2$. In terms of the mode-n product defined before, this matrix product can be rewritten as $\mathbf{A} = \Sigma \times_1 \mathbf{U}_1 \times_2 \mathbf{U}_2$.

By extension, an order $N > 2$ tensor $\mathcal{A}$ is an *N*-mode matrix comprising *N* spaces. "*N*-mode" SVD is an extension of SVD that orthogonalizes these *N* spaces as follows

$$\mathcal{A} = \mathcal{S} \times_1 \mathbf{U}_1 \times_2 \mathbf{U}_2 \ldots \times_N \mathbf{U}_N$$

as illustrated in Figure 3 for the case $N = 3$. Tensor $\mathcal{S}$, known as the core tensor, governs the interaction between the mode matrices $\mathbf{U}_n$, for $n = 1, \ldots, N$.

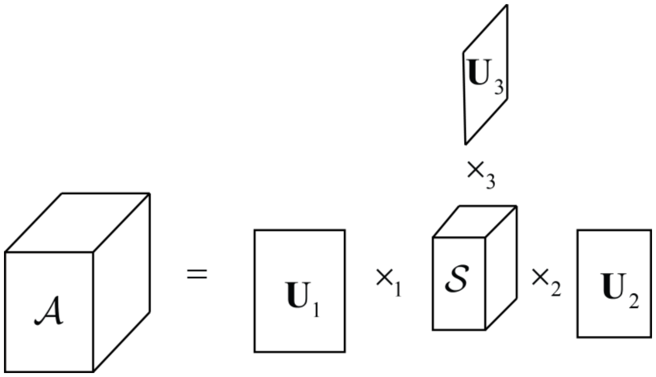

Figure 3. Decomposition of a 3rd –order tensor.

Supplementary References

1. Nolker C, Ritter H: **Visual recognition of continuous hand postures**. *Neural Networks, IEEE Transactions on* 2002, **13**(4):983-994.
2. Tsaig Y, Averbuch A: **Automatic segmentation of moving objects in video sequences: a region labeling approach**. *Circuits and Systems for Video Technology, IEEE Transactions on* 2002, **12**(7):597-612.
3. Deng Y, Manjunath B: **Unsupervised segmentation of color-texture regions in images and video**. *Pattern Analysis and Machine Intelligence, IEEE Transactions on* 2001, **23**(8):800-810.
4. Sobottka K, Pitas I: **Segmentation and tracking of faces in color images**. In: *Automatic Face and Gesture Recognition, 1996, Proceedings of the Second International Conference on: 1996*: IEEE; 1996: 236-241.
5. Faloutsos C, Kolda TG, Sun J: **Mining large time-evolving data using matrix and tensor tools**. In: *ICDM Conference: 2007*; 2007.
6. De Lathauwer L, De Moor B, Vandewalle J: **A multilinear singular value decomposition**. *SIAM journal on Matrix Analysis and Applications* 2000, **21**(4):1253-1278.
7. De Lathauwer L, De Moor B, Vandewalle J: **On the best rank-1 and rank-(R 1, R 2,..., Rn) approximation of higher-order tensors**. *SIAM journal on Matrix Analysis and Applications* 2000, **21**(4):1324-1342.
